# *LATERAL ORGAN BOUNDARIES DOMAIN 25* functions as a key regulator of haustorium development in dodders

**DOI:** 10.1093/plphys/kiab231

**Published:** 2021-05-27

**Authors:** Min-Yao Jhu, Yasunori Ichihashi, Moran Farhi, Caitlin Wong, Neelima R Sinha

**Affiliations:** 1 The Department of Plant Biology, University of California, Davis, California 95616, USA; 2 RIKEN BioResource Research Center, Tsukuba, Ibaraki 305-0074, Japan; 3 The Better Meat Co., West Sacramento, California 95691, USA

## Abstract

Parasitic plants reduce crop yield worldwide. Dodder (*Cuscuta campestris*) is a stem parasite that attaches to its host, using haustoria to extract nutrients and water. We analyzed the transcriptome of six *C. campestris* tissues and identiﬁed a key gene, *LATERAL ORGAN BOUNDARIES DOMAIN 25* (*CcLBD25*), as highly expressed in prehaustoria and haustoria. Gene coexpression networks from different tissue types and laser-capture microdissection RNA-sequencing data indicated that *CcLBD25* could be essential for regulating cell wall loosening and organogenesis. We employed host-induced gene silencing by generating transgenic tomato (*Solanum lycopersicum*) hosts that express hairpin RNAs to target and down-regulate *CcLBD25* in the parasite. Our results showed that *C. campestris* growing on *CcLBD25* RNAi transgenic tomatoes transited to the flowering stage earlier and had reduced biomass compared with *C. campestris* growing on wild-type (WT) hosts, suggesting that parasites growing on transgenic plants were stressed due to insufficient nutrient acquisition. We developed an in vitro haustorium system to assay the number of prehaustoria produced on strands from *C. campestris*. *Cuscuta campestris* grown on *CcLBD25* RNAi tomatoes produced fewer prehaustoria than those grown on WT tomatoes, indicating that down-regulating *CcLBD25* may affect haustorium initiation. *Cuscuta campestris* haustoria growing on *CcLBD25* RNAi tomatoes exhibited reduced pectin digestion and lacked searching hyphae, which interfered with haustorium penetration and formation of vascular connections. The results of this study elucidate the role of *CcLBD25* in haustorium development and might contribute to developing parasite-resistant crops.

## Teaser

Transcription factor CcLBD25 plays a pivotal role in haustorium initiation, regulating pectin digestion, and searching hyphae development during the haustorium penetration process.

## Introduction

Parasitic plants are heterotrophic, reducing the yields of crops worldwide (Agrios, 2005; Yoder and Scholes, 2010). They parasitize host plants using specialized organs known as haustoria, which extract nutrients and water from the hosts. *Cuscuta* species (dodders) are stem holoparasites without functional roots and leaves. Stems of *Cuscuta* spp. coil counterclockwise around their host and then form a series of haustoria along stems to attach to the hosts (Furuhashi et al., 2011; Alakonya et al., 2012). *Cuscuta campestris* is one of the most widely distributed and destructive parasitic weeds (Parker, 2021). A better understanding of the underlying molecular mechanisms of *C. campestris* haustorium development will aid in applying parasitic weed control and producing parasitic plant-resistant crops.

Many previous studies have identified the key factors needed for seed germination, host recognition, and haustorium induction and growth in root parasites (Shen et al., 2006; López-Ráez et al., 2009; Yoder and Scholes, 2010). Focusing on haustorium development, a previous study indicated that root parasitic plants co-opted the mechanism of lateral root formation in haustorium organogenesis (Ichihashi et al., 2017). The LATERAL ORGAN BOUNDARIES DOMAIN (LBD) family of transcription factors (TFs) are reported to be crucial in both lateral root formation in nonparasitic plants and haustorium developmental programming in root parasites (Ichihashi et al., 2020). In nonparasitic model plants, like Arabidopsis, LBD genes are shown to be involved in auxin signaling, interact with AUXIN RESPONSE FACTORs, and promote lateral root formation (Mangeon et al., 2010; Porco et al., 2016). During the haustorium development stage, LBD orthologs are reported to be upregulated at attachment sites of root parasitic plants like *Thesium chinense* (Ichihashi et al., 2017) and *Striga hermonthica* (Yoshida et al., 2019). On the other hand, the molecular pathways regulating haustorium development in stem parasitic plants are still largely unexplored. Although a few gene orthologs that regulate auxin accumulation during lateral root development in nonparasitic plants are found to be expressed in *Cuscuta* seedlings and stems, whether these genes are also involved haustorium formation remains unknown (Ranjan et al., 2014). Our previous studies showed that *SHOOT MERISTEMLESS*-like plays a role in *Cuscuta* spp. haustorium development (Alakonya et al., 2012). These results suggest that *Cuscuta* spp. might have repurposed the shoot developmental programs into haustorium organogenesis, but a recent study indicates that some genes that are involved in root development were also expressed in *Cuscuta australis* prehaustoria and haustoria (Sun et al., 2018), suggesting that the lateral root programming system was co-opted into haustorium development.

In this study, we provide an insight into the gene regulatory mechanisms of haustorium organogenesis and identify one of the LBD TFs, *CcLBD25*, as a vital regulator of *C. campestris* haustorium development. This discovery supports the hypothesis that stem parasitic plants adapted both shoot and root molecular machinery into haustorium formation. Using detailed transcriptome analysis and gene coexpression networks (GCNs) coupled with cellular and developmental phenotype assays, we also show that *CcLBD25* is not only involved in haustorium initiation through auxin signaling, but also participates in other aspects of haustorial developmental reprogramming, including cell wall loosening, searching hyphae development, and other phytohormone mediated signaling pathways. The results of this study will not only shed light on the field of haustorium development in stem parasitic plants but will also help develop a potentially universal parasitic weed-resistant system in crops to reduce economic losses caused by both root and stem parasites.

## Results

### Establishing genomic resources for *C. campestris* and constructing GCNs that regulate haustorium formation

**Figure 1:**
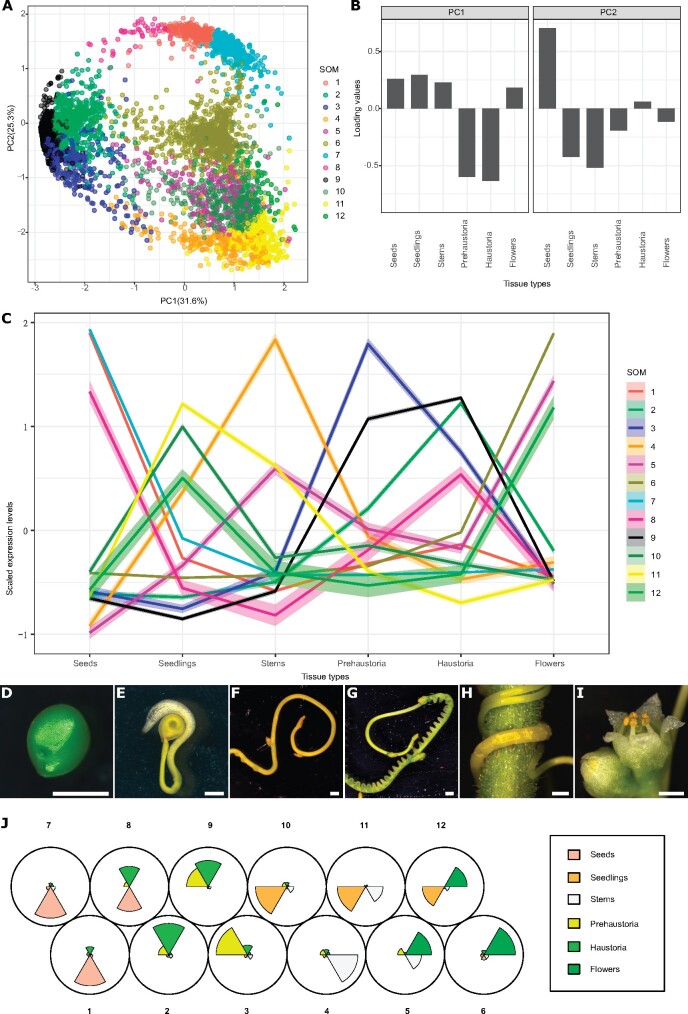
PCA and SOMs clustering of gene expression in *C. campestris* tissue type RNA-seq data mapped to *C. campestris* genome. A, PCA analysis based on gene expression across different *C. campestris* tissues. Each dot represents a gene and is in the color indicating its corresponding SOM group. The percentage next to each axis indicates the proportion of variance that can be explained by the PC. B, Loading values of PC1 and PC2. PC1 separates the genes that are specifically expressed in intrusive tissues (prehaustoria and haustoria) from those that are expressed in nonintrusive tissues. PC2 divides the seed-specific genes from other genes. C, Scaled expression levels of each SOM group across different *C. campestris* tissue types. Each line is colored based on the corresponding SOM groups. The highlighting around the lines indicates a 95% confidence interval. D–I, The six different tissue types that were used in this transcriptomic study. Scale bars = 1 mm. D, Seed. E, Seedling. F, Stem. G, Prehaustoria. H, Haustoria. White arrowheads indicate haustoria. I, Flowers. J, A code plot of SOM clustering based on gene expression in *C. campestris* tissue type RNA-seq data mapped to the *C. campestris* genome. Each sector represents a tissue type and is in the color indicating its corresponding tissue type. The size of each sector illustrates the amount of expression from each tissue type in SOM groups.

In this study, we analyzed the transcriptome of different *C. campestris* tissues, including seeds, seedlings, stems, prehaustoria, haustoria, and flowers, grown on the tomato (*Solanum lycopersicum*) Heinz 1706 (H1706) cultivar and *Nicotiana benthamiana* (Ranjan et al., 2014) by mapping reads to the recently available genome of *C. campestris* (Vogel et al., 2018). In general, seed tissues have distinctively different gene expression profiles compared to all other tissues (Supplemental Figure S1). In addition, the expression patterns in invasive tissues (prehaustoria and haustoria) and noninvasive tissues are also disparate (Supplemental Figure S1). We conducted principal component analysis (PCA) analysis and noticed that the genes that are highly expressed in invasive tissues can be separated from the genes that are highly expressed in noninvasive tissues on PC1 (Figure 1, A and B). To identify the genes that might be involved in haustorium development, we performed clustering analysis using self-organizing maps (SOMs) in R, and identified a cluster enriched with genes that are highly expressed in both prehaustoria and haustoria tissues (SOM9; Figure 1;Supplemental Figure S2; Supplemental Table S1).

**Figure 2:**
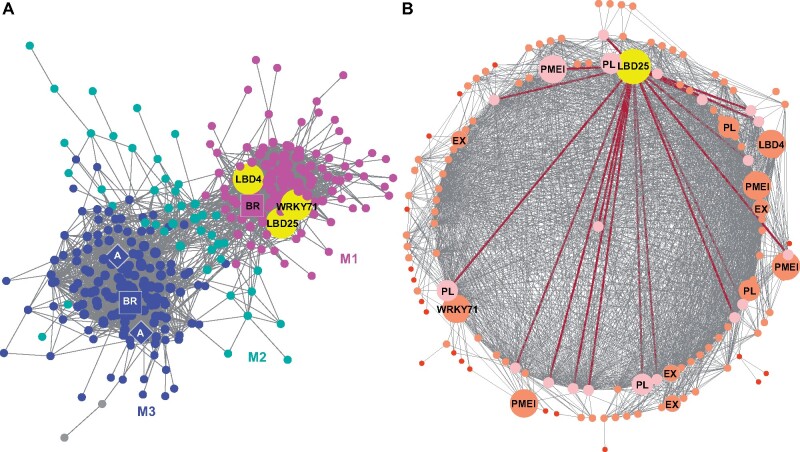
SOM9 GCNs from *C. campestris* tissue type RNA-seq data. A, GCN of genes that are classified in SOM9, which includes genes that are highly expressed in both prehaustoria and haustoria. This SOM9 GCN is composed of three major modules. Magenta indicates genes in Module 1, which has enriched biological process GO term “plant-type cell wall loosening.” Cyan indicates genes in Module 2. Blue indicates genes in Module 3, which has enriched biological process GO terms “transport, response to hormones, secondary metabolite biosynthetic process, and regulation of lignin biosynthetic process.” The only three TFs in Module 1 are enlarged and labeled in yellow. The genes involved in auxin transport are labeled in diamonds. The genes involved in brassinosteroid signaling are labeled in squares. B, GCN of genes that are classified in SOM9 Module 1. Dark red lines indicate the connection between *CcLBD25* (yellow) and its first layer neighbors. The genes that are first layer neighbors of *CcLBD25* are labeled in pink. The genes that are second layer neighbors of *CcLBD25* are labeled in orange with medium size dots. The genes that are outside the second layer neighbors of *CcLBD25* are labeled in red with small size dots. The only three TFs as well as cell wall loosening related genes are enlarged, highlighted and labeled in the network. A and B, PL. A, auxin efflux carrier-like protein. BR, brassinosteroid insensitive 1-associated receptor kinase 1-like.

We focused on the genes contained in this SOM9 cluster and constructed a GCN. Using the fast greedy modularity optimization algorithm to analyze the GCN community structure (Clauset et al., 2004) and visualizing the network using Cytoscape (Cline et al., 2007), we noticed this SOM9 GCN is composed of three major modules (Figure 2A;Supplemental Table S2). Since the current gene annotation of *C. campestris* genome is not as complete as that of most model organisms, we used BLAST to combine our previously annotated transcriptome with current *C. campestris* genome gene IDs (Supplemental Table S3). With this more comprehensive annotation profile, we conducted Gene Ontology (GO) enrichment analysis using the TAIR ID for each *C. campestris* gene in the network to identify the major GO term for each module (Supplemental Table S4). Based on our GO enrichment results, the major biological process of Module 1 can be classified as “plant-type cell wall loosening,” and the cellular component of Module 1 is “extracellular region and intracellular membrane-bounded organelle” (Figure 2; Supplemental Table S4). This result indicates the genes contained in Module 1 are mostly involved in cell wall loosening, which is needed for the haustorium to penetrate through the host tissue. On the other hand, the major biological processes of Module 3 include “transport, response to hormones, secondary metabolite biosynthetic process, and regulation of lignin biosynthetic process.” The molecular function of Module 3 is “transmembrane transporter activity,” and the cellular component of Module 3 is “plasma membrane” (Figure 2A;Supplemental Table S4). This analysis suggests that these genes might be involved in later stages of development and nutrient transport from the host to the parasite once a connection is established between the host and the parasite.

To identify the key regulators in the haustorium penetration process, we focused on genes in Module 1 and calculated the degree centrality and betweenness centrality scores of each gene within this group. Many central hub genes in Module 1 are proteins or enzymes involved in cell wall modifications, like pectin lyases (PLs), pectinesterase inhibitors, and expansins (EXs; Figure 2B;Supplemental Table S2). To find the upstream regulators of these pathways, we focused on TFs that are classified in Module 1. Intriguingly, only three TFs are included in Module 1: LBD gene 25 (*CcLBD25*; Cc019141), *CcLBD4* (Cc017015), and *CcWRKY71* (Cc004070; Figure 2B;Supplemental Table S2). According to the GCN of SOM9, we noticed that *CcLBD25*, *CcLBD4*, and *CcWRKY71* share several common first-layer neighbors (Figure 2). Based on previous reports, *AtLBD25* regulates lateral root development in *Arabidopsis* by promoting auxin signaling (Dean et al., 2004; Mangeon et al., 2010). Furthermore, an *LBD25* ortholog (*TcLBD25*) in *T. chinense*, a root parasitic plant in the Santalaceae family, was also detected to be upregulated during the haustorium development process (Ichihashi et al., 2017). Using *CcLBD25* nucleotide sequence alignments and a *LBD25* phylogenetic tree constructed using MEGA X (Kumar et al., 2018; Supplemental Figure S3; Supplemental Dataset S1), we confirmed orthology between the *TcLBD25*, *AtLBD25*, and *CcLBD25* genes. Based on these serendipitous pieces of evidence, we suspected that *CcLBD25* may also regulate haustorium formation and the parasitism process in *C. campestris*.

**Figure 3:**
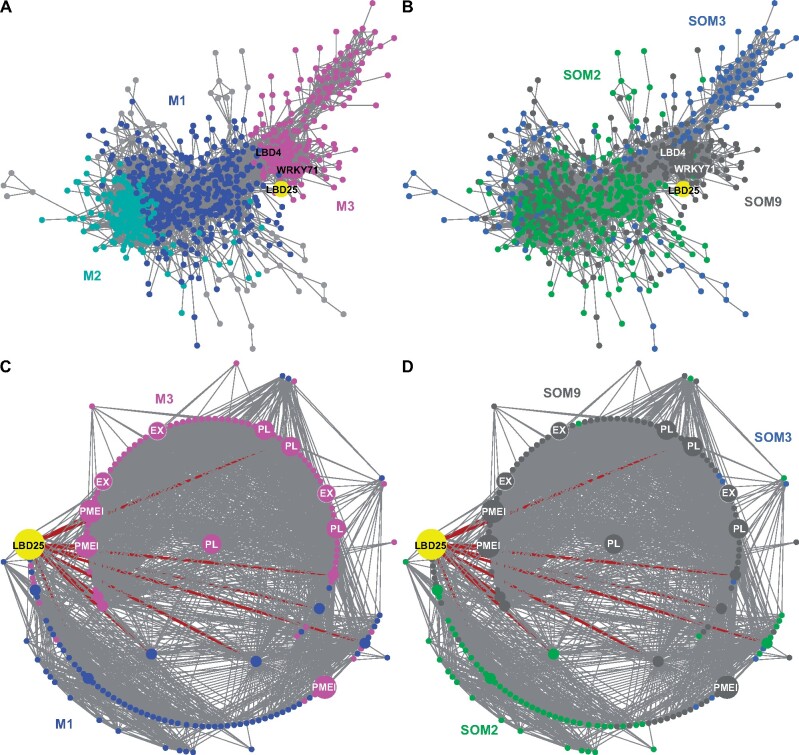
GCNs of SOM2, 3, 9 genes based on *C. campestris* tissue type RNA-seq data. A, GCN of genes that are in SOM2, SOM3, and SOM9 with colors based on network modules. This SOM2+SOM3+SOM9 GCN is composed of three major modules. Blue indicates genes in Module 1, which has the biological process GO enrichment “morphogenesis of a branching structure, plant organ formation, strigolactone responses, and biosynthetic processes.” Cyan indicates genes in Module 2, which has the biological process GO enrichment “response to karrikin, hormone-mediated signaling pathway and defense response.” Magenta indicates genes in Module 3, which has the biological process GO enrichment “plant-type cell wall loosening.” Light grey indicates genes that are not included in Modules 1, 2, or 3. B, GCN of genes that are in SOM2, SOM3, and SOM9, with colors based on SOM clustering groups. Green indicates genes in SOM2. Blue indicates genes in SOM3. Grey indicates genes in SOM9, which includes the genes that are highly expressed in both prehaustoria and haustoria. SOM2 includes genes that are only highly expressed in haustoria and SOM3 includes genes that are only highly expressed in prehaustoria. C, GCN of *CcLBD25* and its first and second layer neighbors with colors based on network modules as in (A). D, GCN of *CcLBD25* and its first and second layer neighbors with colors based on SOM clustering groups as in (B). C and D, Dark red lines indicate the connection between *CcLBD25* and its first layer neighbors. The genes that are second-layer neighbors of *CcLBD25* are labeled with medium size dots. The genes that are outside the second layer neighbors of *CcLBD25* are labeled with small size dots. *CcLBD25* and cell wall loosening related genes are enlarged, highlighted, and labeled in the network.

To understand the role of *CcLBD25* and the potential connection with other genes, we included genes in SOM2 (genes that are only highly expressed in haustoria) and SOM3 (genes that are only highly expressed in prehaustoria) to build a more comprehensive GCN (Figure 1;Supplemental Figure S2). Based on the community structure analysis, this comprehensive network is composed of three major modules (Figure 3A;Supplemental Table S5). Based on our GO enrichment results, the major biological process of Module 3 is plant-type cell wall loosening and the major biological processes of Module 1 include morphogenesis of a branching structure, plant organ formation, and several hormone responses and biosynthetic processes (Supplemental Table S6). *CcLBD25* itself is placed in Module 1 (Supplemental Table S5), but *CcLBD25* has many first layer connections with genes that are classified in Modules 1 or 3 (Figure 3, A and C). This result indicates that *CcLBD25* might play a role in connecting genes involved in different pathways or aspects of haustorium development. Furthermore, by coloring the network with their corresponding SOM groups, we noticed that even though *CcLBD25* itself is in SOM9, many of the *CcLBD25* first and second layer neighbors are in SOM2 and SOM3 (Figure 3, B and D). Thus, *CcLBD25* might be a key regulator of the haustorium development process in both early and late stages of haustorium development and may also play a critical role in coordinating the function of genes that are expressed only during discrete developmental stages.

### Zooming into tissue-specific expression using laser-capture microdissection coupled with RNA-seq

**Figure 4:**
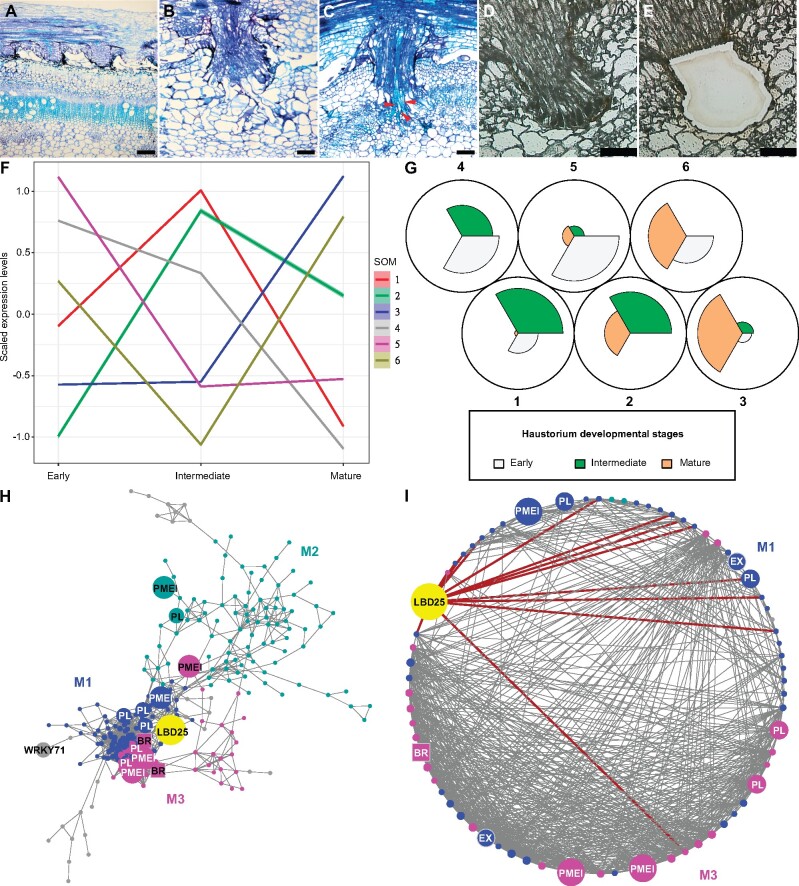
SOM clustering and GCNs of gene expression in *C. campestris* haustoria across three developmental stages (from the LCM RNA-seq data). A–E, Sections of developmental stages for LCM RNA-seq. A–C, Three developmental stages for LCM RNA-seq. Paraffin sections stained with Toluidine Blue. A, Early stage. B, Intermediate stage. C, Mature stage. The red arrowheads indicate vascular connections between host and *C. campestris*. D–E, The *C. campestris* haustorium tissues were collected using LCM. A and C, Scale bars = 250 µm. B, D, and E, Scale bars = 100 µm. F, Scaled expression levels of each SOM group across three haustorium developmental stages. Each line is colored based on the corresponding SOM group. The highlighting around the lines indicates a 95% confidence interval. G, A code plot of SOM clustering illustrating which developmental stages are highly represented in each SOM group by the size of sectors. Each sector represents a developmental stage and is in the color indicating its corresponding developmental stage. H, GCN based on LCM RNA-seq expression profiles with genes in tissue type RNA-seq SOM9. Blue indicates genes in Module 1, which has enriched biological process GO term “plant-type cell wall loosening.” Cyan indicates genes in Module 2, which has enriched biological process GO term “respiratory burst.” Magenta indicates genes in Module 3, which has enriched biological process GO term “brassinosteroid mediated signaling pathway.” Light grey indicates genes that are not included in Modules 1, 2, or 3. *CcLBD25*, *CcWRKY71*, and pectin degradation-related genes are enlarged, highlighted, and labeled in the network. The genes involved in brassinosteroid signaling are labeled in squares. I, GCN of *CcLBD25* and its first and second layer neighbors with colors based on network modules as in (H). Dark red lines indicate the connection between *CcLBD25* and its first layer neighbors. *CcLBD25* and cell wall loosening related genes are enlarged, highlighted, and labeled in the network. H–I, PL.

Our first transcriptome data came from hand-collected tissue samples. To further dissect *Cuscuta* haustorium developmental stages, we used laser-capture microdissection (LCM) with RNA-sequencing (RNA-seq) to analyze only pure haustorial tissues from three different haustorium developmental stages (Figure 4, A–E). Based on a previous study, changes in the levels of jasmonic acid and salicylic acid are observed ∼36–48 h after first haustorial swelling, which is ∼4 d postattachment (DPA; Runyon et al., 2010). We also noticed that haustorium growth is a continuous process for *C. campestris*, so all developmental stages of haustoria can be found on the same strand at the 4 DPA time point. Therefore, we focused on 4 DPA and defined three developmental stages based on their haustorium structure: early (the haustorium has just contacted the host), intermediate (the haustorium has developed searching hyphae but has not formed vascular connections), and mature (a mature haustorium with continuous vasculature between host and parasite; Figure 4, A–C). *Cuscuta campestris* haustorium tissues, especially the protruding region of haustoria, were collected from *C. campestris* using LCM at these three developmental stages attached to H1706 and subjected to RNA-seq (Figure 4, D–E).

Next, we mapped our LCM RNA-seq data to the *C. campestris* genome. Visualizing the gene expression changes using multidimensional scaling (MDS) showed that the expression profile of the mature stage is distinct from the early and intermediate stages (Supplemental Figure S4). We then conducted clustering analyses using SOM to group genes based on their expression patterns at these three different developmental stages (Figure 4, F–G). According to our PCA analysis, PC1 obviously separated genes that are specifically expressed in the mature stage from those expressed in the other two stages, and PC2 distinguished the genes expressed in the early stage from those expressed in the intermediate stage (Supplemental Figure S5). Interestingly, and similar to what was seen in our tissue type transcriptome data, *CcLBD25* is grouped in SOM6, which is the cluster of genes that are relatively highly expressed in both early and mature stages (Figure 4, F–G;Supplemental Figure S6; Supplemental Table S7). Notably, *CcLBD25* expression in intermediate-stage is relatively reduced compared with early-stage or mature-stage but is still much higher compared with other noninvasive tissues like seed, seedlings, stems, and flowers. Genes with low expression levels were often not detectable in LCM RNA-seq data, which might be caused by the preparation process of LCM tissues, including fixation, sectioning, and dissection processes, which are likely to lead to loss of some RNAs due to unpreventable degradation. To investigate gene regulatory dynamics within the haustorium developmental process, we used the same gene list from tissue type RNA-seq SOM9 and constructed another GCN of these genes that was based on the LCM RNA-seq expression profiles (Figure 4H). By using the same gene list, but the expression dataset from samples of precisely collected haustorial cells, we obtained detailed regulatory connections between genes by comparing the tissue type GCN and LCM GCN (Figures 2A, 4H). Based on the fast greedy community structure analysis, this LCM GCN is composed of three major modules with *CcLBD25* in Module 1 (Figure 4H;Supplemental Table S8). According to our GO enrichment results, the major biological process for Module 1 is plant-type cell wall loosening, and for Module 3, is brassinosteroid mediated signaling pathway (Figure 4H;Supplemental Table S9). In addition to cell wall loosening related enzyme encoding genes forming central hubs, we noticed *CcLBD25* is the TF with the highest number of connections in Module 1. *CcLBD25* has 13 first layer neighbors and 70 second-layer neighbors, including many cell wall loosening-related genes (Figure 4, H–I;Supplemental Table S8). Zooming in to focus on *CcLBD25*, we noticed that the *CcLBD25* first and second layer neighbors are genes classified in Modules 1 or 3, indicating that *CcLBD25* might play a role in connecting these two pathways. Many of the *CcLBD25* first and second layer neighbors are pectin degradation-related genes like *PL* and *PMEI*. On the other hand, *CcLBD4* is not in the LCM GCN, and *CcWRKY71* is at a marginal location with only one connection. This result provided further support for our hypothesis that *CcLBD25* is the major TF regulating cell wall modification in the haustorium penetration process. *CcLBD4* and *CcWRKY71* might also be key regulators but are likely involved in a different aspect of haustorium development. Thus, we focused our attention on understanding the function of *CcLBD25* in haustorium development.

### Cross-species RNAi (host-induced gene silencing) *CcLBD25* affects whole-plant phenotypes and reduces parasite fitness

**Figure 5:**
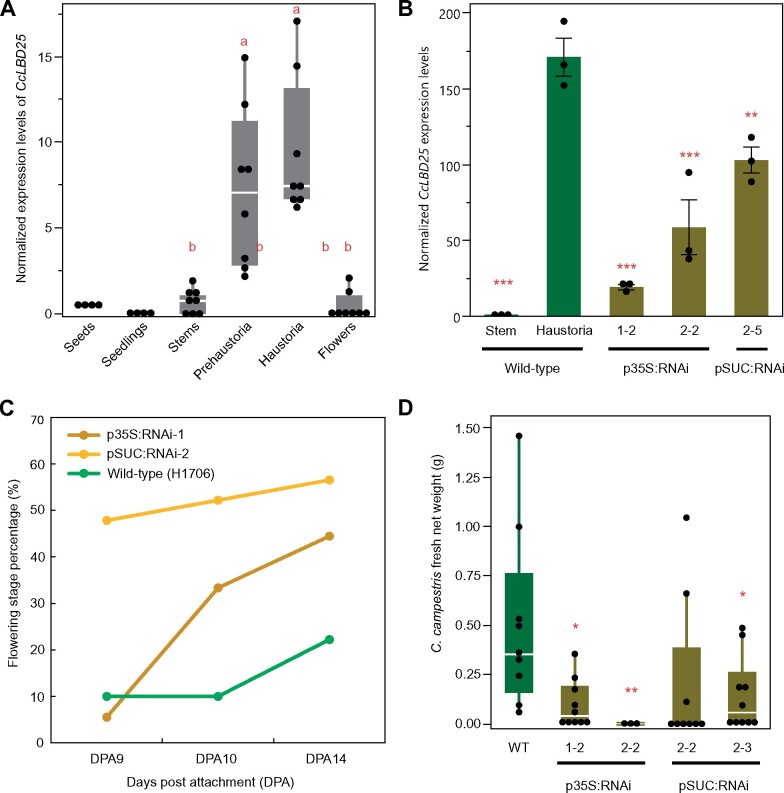
Gene expression levels and whole-plant phenotypes of *C. campestris* growing on HIGS *CcLBD25* RNAi transgenic plants. A, The normalized expression level of *CcLBD25* in six different tissue types of *C. campestris* (from RNA-seq data). Data presented are assessed using pair-wise comparisons with the Tukey’s test. *P*-values of the contrasts between “a” and “b” are ˂0.001. B, Expression levels of *CcLBD25* in *C. campestris* haustoria grown on WT tomatoes and T2 *CcLBD25* RNAi transgenic plants (by RT-qPCR). Data presented are assessed using one-tailed Welch’s *t* test with WT haustoria as control. *P-value < 0.05; **P-value < 0.01; ***P-value < 0.005. The error bars indicate standard errors of the data. All data points are plotted as black dots. C, The flowering time of *C. campestris* growing on WT tomatoes and T1 *CcLBD25* RNAi transgenic plants. The early transition to the ﬂowering stage indicates that *C. campestris* may be growing under stress conditions because they might not obtain sufficient nutrients from their host. Quantification was assessed with the whole plant as a unit. Sample size: WT, 9 biological replicates; p35S:RNAi-1, 18 biological replicates; pSUC:RNAi-2, 23 biological replicates. D, Biomass of *C. campestris* growing on WT tomatoes and T2 *CcLBD25* RNAi transgenic plants. Fresh net weights of *C. campestris* were measured in grams. Data presented are assessed using one-tailed Welch’s *t* test with WT as control. *P-value < 0.05; **P-value < 0.01. B–D, p35S:RNAi indicates the transgenic plants with the 35S promoter driving the *CcLBD25* RNAi construct. pSUC:RNAi indicates the transgenic plants with the SUC2 promoter driving the *CcLBD25* RNAi construct. A and D, The centerline in the box indicates the median. The bottom and top of the box indicate the 25th and 75th quantiles. The whiskers represent the expected variation of the data. The whiskers extend 1.5 times the interquartile range from the top and bottom of the box. All measured data points are plotted as black dots.

In our previous studies, we found cross-species transport of mRNAs and siRNAs between *C. campestris* and their hosts (when the initial haustoria are successfully connected with host vascular tissue), and demonstrated host-induced gene silencing (HIGS; Runo et al., 2011; Alakonya et al., 2012). Many previous studies have also shown that large-scale mRNA and small RNAs are transported through the haustorium connections in *Cuscuta* species (Kim et al., 2014; Johnson et al., 2019). Therefore, we generated transgenic host tomatoes with hairpin RNAs that target and down-regulate *CcLBD25* after the parasite forms the first attachment and takes up RNAs from the host (Supplemental Figure S7). When *C. campestris* grows on wild-type (WT) tomato hosts, *CcLBD25* is highly expressed in invasive tissues (Figure 5, A and B). However, *CcLBD25* expression levels are significantly knocked down in the tissues on and near the initial functional attachment sites of *C. campestris* plants that are growing on *CcLBD25* RNAi transgenic plants (Figure 5B). Based on our results, expressing *CcLBD25* RNAi constructs has no effect on tomato plant growth and, with one exception, transgenic *CcLBD25* RNAi tomato plants have the same phenotype as WT plants (Supplemental Figure S8). Notably, *CcLBD25* and *SlLBD25* nucleotide sequences shared low similarity based on results of Blastn with the most recently published tomato genome (ITAG 4.0; Supplemental Data S1). This also explains why using *CcLBD25* RNAi constructs do not influence tomato growth (Supplemental Figure S8). We suspect that the p35S:RNAi line 2-2 might be different from WT due to tissue culture or insertion effects. Nevertheless, pSUC:RNAi would be a better choice for agricultural application due to the specific expression of the RNAi construct in phloem tissues, which would allow efficient transfer of siRNAs to *C. campestris* after establishment of vascular connection. However, for the purpose of verifying the function of *CcLBD25* in *C. campestris*, both p35S:RNAi lines and pSUC:RNAi lines worked well and successfully downregulated the expression of *CcLBD25* in *C. campestris* (Figure 5B). If *CcLBD25* is important in haustorium development and parasitism, then downregulating *CcLBD25* should influence haustorium structure or formation, and might also affect nutrient transport. To verify our hypothesis, we measured flowering time in *C. campestris* growing on various tomato hosts. The result showed that parasites growing on *CcLBD25* RNAi transgenic tomatoes transitioned to the flowering stage and subsequently senesced earlier than those growing on WTs (Figure 5C). Based on previous studies, many plant species respond to environmental stress factors by inducing flowering (Wada and Takeno, 2010; Riboni et al., 2014). This early transition to the reproductive stage and senescence in *C. campestris* grown on *CcLBD25* RNAi plants suggests that *C. campestris* was growing under stress, likely because of nutrient deficiency.

To verify if downregulating *CcLBD25* affects the ability of the parasite to acquire resources from the host, we also measured the biomass of *C. campestris* grown on WT H1706 and *CcLBD25* RNAi transgenic plants. At 14 DPA, we noticed that *C. campestris* plants grown on *CcLBD25* RNAi transgenic tomatoes had less biomass compared with the *C. campestris* plants grown on WT H1706 (Figure 5D). Both whole-plant level phenotypes suggest that *CcLBD25* might be involved in haustorium development and knocking down the expression level of *CcLBD25* influences the ability of *C. campestris* to establish connections with hosts and interferes with parasite nutrient acquisition.

### Using an in vitro haustoria system to investigate the impact of *CcLBD25* on early-stage haustorium development

**Figure 6:**
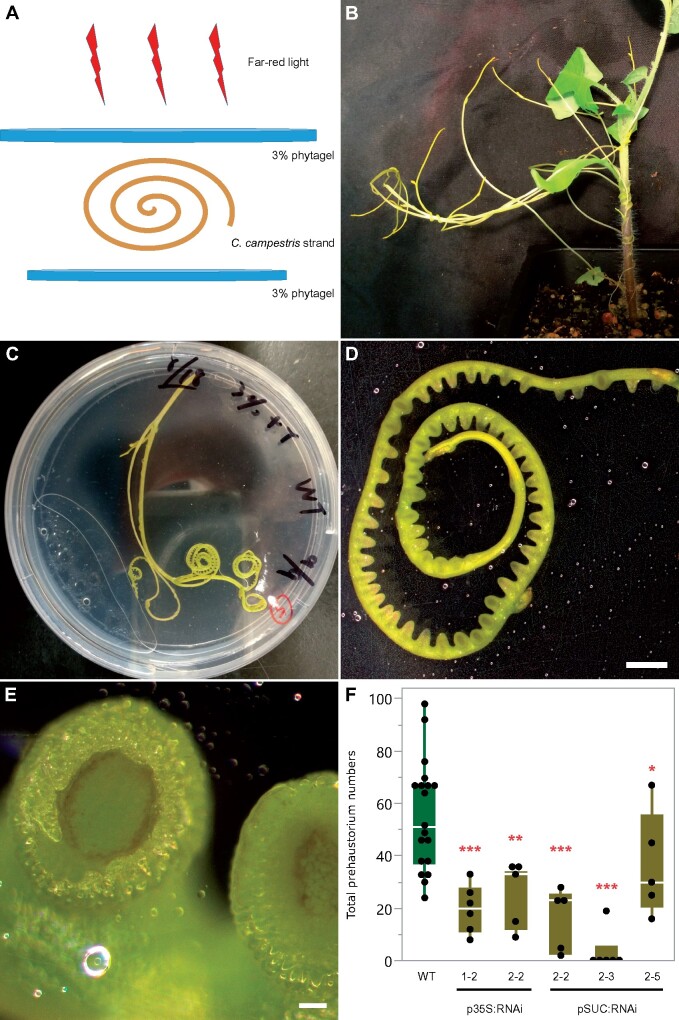
Far-red light-induced IVH phenotypes of *C. campestris* growing on T2 HIGS *CcLBD25* RNAi transgenic plants. A, An illustration of the setup for the IVH system. Each *C. campestris* strand that was used in the IVH system is ∼10 cm long. B, *Cuscuta campestris* strands near the haustorium attachment sites. C, An IVH plate with a *C. campestris* strand sandwiched in between two layers of agar to provide sufficient physical contact signals. D and E, After illuminating these plates under far-light for 5 d, prehaustoria is readily visible. D, Scale bar = 2 mm. E, Scale bar = 100 µm. F, *C. campestris* strands were detached and subjected to IVH, and the numbers of prehaustoria were counted. Data presented are assessed using one-tailed Welch’s *t* test with WT as control. *P-value < 0.06; **P-value < 0.001; ***P-value < 0.0005. p35S:RNAi indicates the transgenic plants with the 35S promoter driving the *CcLBD25* RNAi construct. pSUC:RNAi indicates the transgenic plants with the SUC2 promoter driving the *CcLBD25* RNAi construct. The centerline in the box indicates the median. The bottom and top of the box indicate the 25th and 75th quantiles. The whiskers represent the expected variation of the data. The whiskers extend 1.5 times the interquartile range from the top and bottom of the box. All measured data points are plotted as black dots.

Previous studies indicate that several auxin-inducible *LBD* genes function in lateral root initiation (Goh et al., 2012). We noticed that auxin efflux carriers and auxin-responsive genes are also in the SOM9 GCN (Figure 2). Therefore, we proposed that *CcLBD25* might regulate early-stage haustorium development in *C. campestris*. In order to assay the role of *CcLBD25* in *C. campestris* haustorium initiation, we developed an in vitro haustorium (IVH) system coupled with HIGS (Figure 6A). This method is inspired by the previous discovery that *Cuscuta* haustoria can be induced by physical contact and far-red light signals (Tada et al., 1996) and many studies confirmed that small RNAs and mRNAs can move cross-species through the haustorial phloem connection (David-Schwartz et al., 2008; Alakonya et al., 2012; Kim et al., 2014; Johnson et al., 2019). Therefore, we took the *C. campestris* strands growing near the haustorium attachment sites on WT and *CcLBD25 RNAi* transgenic tomato (Figure 6B) and sandwiched these strands between two layers of agar to provide sufficient physical contact (Figure 6, A and C). We then illuminated these plates under far-red light for 5 d, at which point prehaustoria are readily visible (Figure 6, D–E). Since the IVH induction is rapid and these prehaustoria can easily be separated from the agar, this method allowed us to count prehaustoria numbers under the microscope and validate the effect of *CcLBD25* RNAi on haustorium initiation. The strands from the *C. campestris* grown on *CcLBD25 RNAi* transgenic tomatoes produced much fewer prehaustoria than the strands from those grown on WT (Figure 6F). This result indicates that reduced *CcLBD25* expression impeded haustorium initiation and confirms that *CcLBD25* is a key regulator of early-stage haustorium development, as suggested by our LCM RNA-seq analysis results.

### Down-regulation of *CcLBD25* leads to structural changes in haustoria

**Figure 7:**
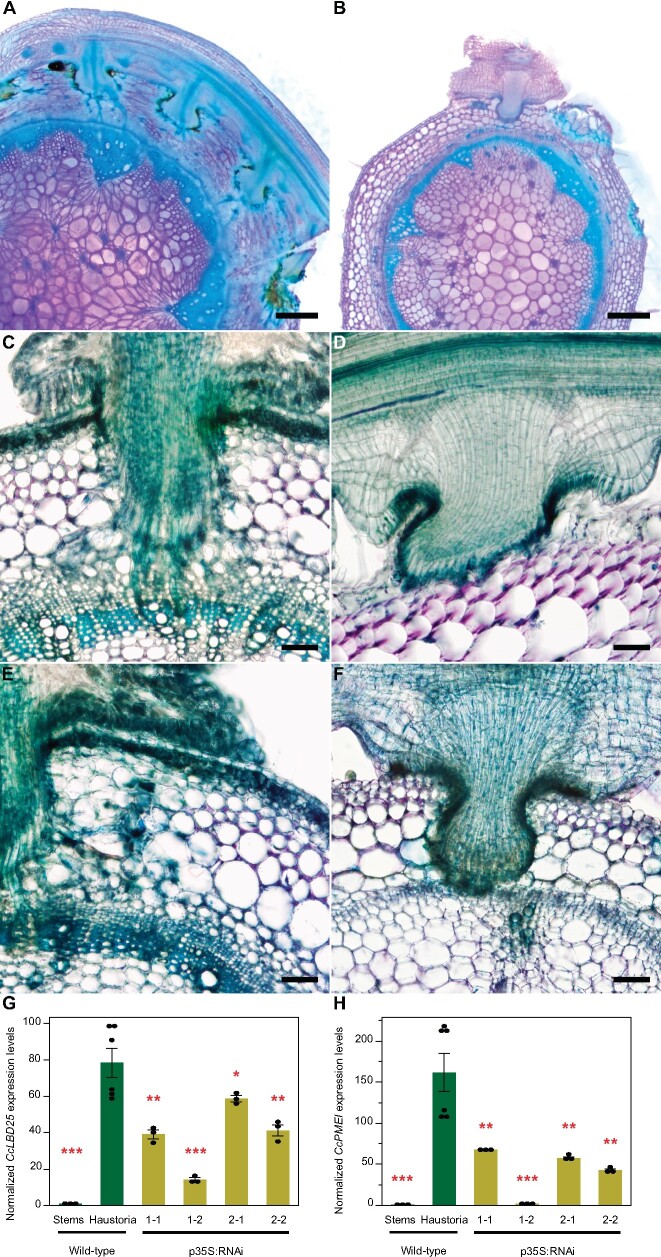
Haustorium phenotypes and gene expression levels of *C. campestris* growing on HIGS *CcLBD25* RNAi transgenic plants. A, C, and E, *Cuscuta campestris* haustoria growing on a WT H1706 host. B, D, and F, *Cuscuta campestris* haustoria growing on T2 *CcLBD25* RNAi transgenic tomato plants. B, p35S:RNAi line 1-1. D, pSUC:RNAi line 1-2. F, p35S:RNAi line 1-2. A, B, Scale bars = 500 µm. C**–**F, Scale bars = 100 µm. A–F, 100 µm thick vibratome sections of fresh haustorium stained with Toluidine Blue O. G, H, Expression levels of *CcLBD25* and *CcPMEI* in *C. campestris* haustoria grown on WT tomatoes and *CcLBD25* RNAi transgenic plants. Data presented are assessed using one-tailed Welch’s *t* test with WT haustoria as control. *P-value < 0.05; **P-value < 0.005; ***P-value < 0.001. p35S:RNAi indicates the transgenic plants with the 35S promoter driving *CcLBD25* RNAi construct. The error bars indicate standard errors of the data.

To verify the crucial role *CcLBD25* plays in haustorium development and to investigate how down-regulating *CcLBD25* affects haustorium structure and the parasitism process, we prepared 100 µm-thick fresh haustorium sections using a vibratome, and stained them with Toluidine Blue O (O’Brien et al., 1964). In sections of haustoria growing on WT plants, we could observe searching hyphae that had penetrated the host cortex region and transformed into xylic or phloic hyphae as they had connected to host xylem and phloem (Figure 7, A, C, and E). However, we observed that many haustoria growing on *CcLBD25* RNAi transgenic tomatoes had formed a dome shape structure and lacked searching hyphae (Figure 7, B, D, and F; Supplemental Figure S9; Supplemental Tables S10 and S11). This result indicates that *CcLBD25* might be involved in development of searching hyphae. Therefore, knocking down of *CcLBD25* affects the ability of *C. campestris* to establish connections with the host vascular system and leads to nutrient deficiency, as observed in the whole-plant level phenotypes.

We also noticed the down-regulation of *CcLBD25* influenced the parasite penetration process. Fresh tissue sections of the haustoria growing on WT showed a clear zone in tomato cortex tissues near haustorium tissues (Figure 7, A, C, and E). Since the metachromatic staining of Toluidine Blue O is based on cell wall composition and pH values and a pink to purple color indicates pectin presence, this result indicates that the pectins in tomato cortex tissues may have been digested or the pH condition in the cell wall had been changed in the haustorium penetration process (Figure 7, A, C, and E). On the other hand, the *C. campestris* growing on *CcLBD25* RNAi transgenic tomatoes still showed pink to purple color in the cortex near the haustorium attachment sites (Figure 7, B and D). Hence, less pectin digestion or cell wall modification happened in tomato cortex tissues near these *CcLBD25* downregulated haustorium tissues compared to the haustoria growing on WT. These haustorium structural phenotypes correspond well with our SOM9 GCN from the *C. campestris* tissue transcriptome. *CcLBD25* is one of the TF central hub genes in Module 1, with many first and second layers of connection to genes involved in cell wall modification, including PLs and pectin methyl-esterase (PME) inhibitors (PMEIs). Based on many previous studies, the interplay between PME and PMEI is an important determinant of cell wall loosening, strengthening, and organ formation (Wormit and Usadel, 2018). Therefore, we hypothesized that PMEIs might be one of the key regulators that cause the haustorium phenotype in *CcLBD25-*downregulated haustorium tissues. To test if the downregulation of *CcLBD25* would affect *PMEI* expression levels, we conducted qPCR to detect *CcPMEI* expression levels in the tissues of *C. campestris* plants that are growing on *CcLBD25* RNAi transgenic plants. Our results show that *CcPMEI* expression levels are also significantly reduced when *CcLBD25* is knocked down (Figure 7, G and H). Thus, *CcLBD25* might directly or indirectly regulate *CcPMEI* at the transcriptional level. These results verify the hypothesis that *CcLBD25* plays an important role in haustorium development and might regulate cell wall modification.

## Discussion

In this study, we demonstrate that *CcLBD25* is a crucial regulator of several aspects of *C. campestris* haustorium development, including haustorium initiation, cell-wall loosening, and searching hyphae growth. We use transcriptome of six *C. campestris* tissue types and RNA-seq data of LCM captured haustoria at three developmental stages to reveal the potential molecular mechanisms and the complexity of gene networks that are regulated during the haustorium formation process. Our results provide a comprehensive analysis of the *CcLBD25* centered regulatory system and illustrate that *CcLBD25* might directly or indirectly coordinate different groups of genes that are expressed only at the early or mature stage during haustorium development.

### Lateral root development and haustorium development

In nonparasitic plants, like Arabidopsis, *AtLBD25* was also named *DOWN IN DARK AND AUXIN1* because *lbd25* mutant plants exhibited reduced sensitivity to auxin and reduced number of lateral roots (Mangeon et al., 2010). These phenotypes indicate that *AtLBD25* functions in lateral root formation by promoting auxin signaling (Mangeon et al., 2010). In the root parasitic plant, *T. chinense*, *TcLBD25* was highly expressed during haustorium formation (Ichihashi et al., 2017). This supports the hypothesis that root parasitic plants co-opted the lateral root formation machinery into haustorium organogenesis. However, whether rootless stem parasitic plants *Cuscuta* spp. also followed the same path to generating haustoria was unknown. In this study, we identified *CcLBD25* as playing a key role in *Cuscuta* haustorium development. Our SOM9 GCN shows that auxin efflux carriers and auxin-responsive genes are also remotely connected with *CcLBD25*, but not in the first or second layers of neighbors (Figure 2). Our hypothesis is that the increased expression of *CcLBD25* might induce the genes that are involved in auxin signaling, which was observed in the Arabidopsis lateral root development system (Dean et al., 2004; Mangeon et al., 2010). These pieces of evidence suggest that *Cuscuta* spp. adapted not only the shoot developmental programs (Alakonya et al., 2012), but also the lateral root programming system, into haustorium organogenesis. According to our *LBD25* gene phylogenetic tree, *CcLBD25* and *TcLBD25* likely evolved convergently to function in haustorium development (Supplemental Figure S3; Supplemental Data S1).

### Development of searching hyphae

Down-regulating *CcLBD25* reduced searching hyphae formation (Figure 7, B, D, and F; Supplemental Figure S9), indicating that *CcLBD25* is involved in searching hyphae development. Surprisingly, *AtLBD25* is not only expressed in roots but is also expressed in pollen (Mangeon et al., 2010). Previous reports indicate that *AtLBD25* is especially highly expressed during the pollen late developmental stage (Kim et al., 2016). Intriguingly, many genes that are involved in haustoria development also play important roles in flower and pollen development (Yang et al., 2015; Yoshida et al., 2019). Recent research on haustoria 3D structures also indicates that the growth pattern of intrusive cells is similar to the rapid polar growth of pollen tubes (Masumoto et al., 2021). Taken together with our results in this study and previous findings in other organisms, we suggest that the genes that regulate pollen development or pollen tube growth, like *LBD25*, might be adapted by parasitic plants for development of haustorium intrusive cells and searching hyphae. This discovery also confirmed the hypothesis that parasitic plants co-opted the developmental reprogramming process from multiple sources instead of just a single organ.

### Cell adhesion and cell wall loosening in parasitism

The mechanical properties and chemical conditions of cell walls have been reported to be critical for regulating plant organ morphogenesis (Chebli and Geitmann, 2017; Zhao et al., 2018). By remodeling cell wall composition or extracellular environments, plants generate local cell wall loosening and strengthening, which allows anisotropic growth processes to occur (Chebli and Geitmann, 2017). Recent studies also indicate that the interaction between pectin and other cell wall components is an important determinant for plant organogenesis (Chebli and Geitmann, 2017; Saffer, 2018), and the interplay between PME and PMEI plays a vital role in regulating physical properties of the cell wall (Wormit and Usadel, 2018). In the root parasitic plant, *Orobanche cumana*, a PME is shown to be present at the host and parasite interface and to have pectolytic activity (Losner-Goshen et al., 1998). These results suggest that parasitic plants produce PME to degrade pectin in the host cell wall and help with haustorium penetration. Our SOM9 GCNs shows that *CcLBD25* is coexpressed with many PLs and PMEIs (Figures 2B; 3, C–D; 4, H–I), implying that *CcLBD25* might be the key TF regulating expression of the enzymes involved in pectin remodeling. The haustoria grown on *CcLBD25* RNAi transgenic plants failed to penetrate host tissues and were unable to create a clear zone at the host and parasite interface (Figure 7, A–F), supporting the existence of a tight connection between *CcLBD25* and pectin-modifying enzymes. *CcLBD25* and PMEIs were coexpressed in the mature stage of haustorium (Figure 4, H–I), when cell wall loosening occurs for haustorium penetration.

On the other hand, since the patterns of demethylesteriﬁcation on homogalacturonans determine cell wall loosening or strengthening, pectin properties also play a role in cell adhesion, which is regulated by PME and PMEI (Wormit and Usadel, 2018). Previous studies also indicate that *Cuscuta* spp. secrete pectin-rich adhesive materials to help with adhesion and allow attachment to their hosts (Vaughn, 2002; Shimizu and Aoki, 2019). This is consistent with our discovery that both *CcLBD25* and *PMEI*s are highly expressed in the early stage of haustorium development, which would be responsible for the adhesion process in *C*. *campestris* (Figure 4, H–I).

## Conclusions

Our detailed bioinformatic analysis on previously published *C. campestris* tissue type transcriptome coupled with LCM of RNA-seq data from three haustorium developmental stages helped us discern the molecular mechanism of parasitic plant haustorium development. The discovery that *CcLBD25* plays a pivotal role in many aspects of haustorium formation shows that the regulatory machinery of haustorium development is potentially shared by both root and stem parasites. Although previous studies have indicated that parasitic plants evolved independently in about 13 different families, this conserved molecular mechanism supports the hypothesis that stem parasitic plants also adapted the lateral root formation programming of nonparasitic plants into haustorium development. The results of this study not only provide an insight into molecular mechanisms by which LBD25 may regulate parasitic plant haustorium development, but also raise potential for developing a universal parasitic weed-resistant crop that can defend against both stem and root parasitic plants at the same time.

## Materials and methods

### *Cuscuta campestris* materials

We thank W. Thomas Lanini for providing dodder seeds collected from tomato field in California. These dodder materials were previously identified as *Cuscuta pentagona* (Yaakov et al., 2001), a species closely related to *C. campestris* (Costea et al., 2015). We used molecular phylogenetics of plastid *trnL-F* intron / spacer region, plastid ribulose-1,5-bisphosphate carboxylase/oxygenase large subunit, nuclear internal transcribed spacer, and nuclear large-subunit ribosomal DNA sequences (Stefanović et al., 2007; García et al., 2014; Costea et al., 2015) to verify our dodder isolate is the same as *C. campestris* 201, Rose 46281 (WTU ) from California, USA (Jhu et al., 2020) by comparing with published sequences (Costea et al., 2015).

### RNA-seq data mapping and processing

For *C. campestris* tissue type RNA-seq analysis, we used the raw data previously published (Ranjan et al., 2014). This RNA-seq data contain six different *C. campestris* tissues, including seeds, seedlings, stems, prehaustoria, haustoria, and flowers, grown on the tomato (*Solanum lycopersicum*) H1706 cultivar and *N. benthamiana*. We mapped both *C. campestris* tissue type and LCM RNA-seq data to the genome of *C. campestris* (Vogel et al., 2018) with Bowtie 2 (Langmead and Salzberg, 2012) and used EdgeR (Robinson et al., 2009) to get normalized trimmed mean of *M* values for further analysis.

### MDS and PCA with SOM clustering

After normalization steps, we used cmdscale in R stats package to create MDS data matrix and then generate MDS plots. For *C. campestris* tissue types RNA-seq data, we selected genes with coefﬁcient of variation >0.85 for PCA analysis. We calculated PC values using prcomp function in R stats package. Selected genes are clustered for multilevel six-by-two hexagonal SOM using som function in the Kohonen package (Wehrens and Buydens, 2007). We visualized the SOM clustering results in PCA plots. The complete gene lists for all SOM units in *C. campestris* tissue type RNA-seq data with SOM distances and PCA PC values are included in Supplemental Table S1. For *C. campestris* LCM RNA-seq data, genes in the upper 50% quartile of coefﬁcient of variation were selected for further analysis. Selected genes were then clustered for multilevel three-by-two hexagonal SOM. The complete gene lists for all SOM units in LCM RNA-seq data with SOM distances and PCA PC values are included in Supplemental Table S7.

### Construct GCNs

We used the genes that are classified in selected SOM groups to build GCNs. The R script is modified from our previously published method (Ichihashi et al., 2014) and the updated script is uploaded to GitHub and included in code availability. The SOM9 GCNs for *C. campestris* tissue type data was constructed with normal quantile cutoff = 0.93. The SOM2 + 3 + 9 GCNs for *C. campestris* tissue type data was constructed with normal quantile cutoff = 0.94. For the GCN of *C. campestris* LCM data, we used the SOM9 gene list from tissue type RNA-seq and constructed the GCN of these genes based on the expression profiles in LCM RNA-seq data with normal quantile cutoff = 0.94. These networks were then visualized using Cytoscape version 3.8.0.

### Functional annotation and GO enrichment analysis of RNA-seq data

Since many genes are not functionally annotated in the recently published *C. campestris* genome (Vogel et al., 2018), we used BLASTN with 1e-5 as an e-value threshold to compare our previously annotated transcriptome final contigs with current *C. campestris* genome genes and only kept the highest scored hit for each gene (Supplemental Table S3). After we obtained this master list, we combined the functional annotation of our published transcriptome based on the NCBI nonredundant database and TAIR10 (Ranjan et al., 2014) with the *C. campestris* genome gene IDs to create a more complete functional annotation (Supplemental Table S9). TAIR ID hits were used for GO Enrichment Analysis on http://geneontology.org/ for gene clusters and modules.

### LCM RNA-seq library preparation and sequencing

We infested approximately four-leaves-stage H1706 tomato plants with *C. campestris* strands. Tomato stems with haustoria were collected at 4 DPA and fixed in formaldehyde–acetic acid–alcohol (FAA). These samples were dehydrated by the ethanol series and embedded in paraffin (Paraplast X-Tra; Thermo Fisher Scientific, Waltham, MA, USA). We prepared 10 μm thick sections on a Leica RM2125RT rotary microtome. Approximately 30 regions of 10 um thickness each were cut from each slide, and three to four slides used per library preparation. Tissue was processed within one month of fixation to ensure RNA quality. Haustorial tissues of the three defined developmental stages were dissected on a Leica LMD6000 Laser Microdissection System. Tissue was collected in lysis buffer from RNAqueous-Micro Total RNA Isolation Kit (Ambion, Austin, TX, USA) and stored at −80**°**C. RNA was extracted using RNAqueous-Micro Total RNA Isolation Kit (Ambion) and amplified using WT-Ovation Pico RNA Amplification System version 1.0 (NuGEN Technologies Inc., San Carlos, CA, USA) following manufacturer instructions. RNA-seq libraries for Illumina sequencing were constructed following a previously published method (Kumar et al., 2012) with slight modifications. Libraries were quantified, pooled to equal amounts, and their quality was checked on a Bioanalyzer 2100 (Agilent, Santa Clara, CA, USA). Libraries were sequenced on a HiSeq2000 Illumina Sequencer at the Vincent J Coates Genomics Sequencing Laboratory at UC Berkeley.

### *CcLBD25* RNAi transgenic plants and HIGS efficiency verification

We used the pTKO2 vector (Snowden et al., 2005; Brendolise et al., 2017), which enables streamlined cloning by using two GATEWAY cassettes positioned at opposite directions, separated by an Arabidopsis ACT2 intron, and under the control of the 35S constitutive promoter. We have previously shown that producing the RNAi construct in phloem cells specifically using the SUC2 promoter was effective for dodder HIGS (Alakonya et al., 2012). Therefore, we replaced the 35S promoter with the SUC2 promoter and generated pTKOS (Supplemental Figure S7). We used BLAST to identify a 292 bp fragment that was specific to *CcLBD25* and different from tomato genes (Supplemental Dataset S2). This RNAi fragment was amplified from *C. campestris* gDNA, TOPO cloned into pCR8/GW-TOPO (Life Technologies, Carlsbad, CA, USA) and LR recombined into pTKO2 and pTKOS (Supplemental Dataset S3). These constructs were then sent to the UC Davis Plant Transformation Facility to generate *CcLBD25* RNAi transgenic tomato plants.

All T0 transgenic plants were selected by kanamycin resistance and their gDNAs were extracted and PCR performed to verify they contained *CcLBD25* RNAi constructs. To validate HIGS efficiency and quantify the expression level of *CcLBD25* and *CcPMEI* in *C. campestris*, dodder tissues were harvested from both *C. campestris* grown on WT plants and T2 *CcLBD25* RNAi transgenic plants. For validating the downregulation of *CcLBD25*, we collected the stem segment with haustoria and prehaustoria at the initial attachment site from *C. campestris* grown on WT and RNAi transgenic plants. About 100 mg tissues were used for each RNA extraction for each genotype sample. We froze tissues in liquid nitrogen and ground them in extraction buffer using a bead beater (Mini Beadbeater 96; BioSpec Products). Following our previously published poly-A-based RNA extraction method (Townsley et al., 2015), we obtained total mRNA from *C. campestris* and then used Superscript III reverse transcriptase (Invitrogen, Carlsbad, CA, USA) for reverse transcription to synthesize cDNA as described by the manufacturer instructions. Real-time qPCR was performed using a Bio-Rad iCycler iQ real-time thermal cycler with Bio-Rad IQ SYBR Green super mix. The sequences of qPCR primer pairs are included in Supplemental Table S12.

### Whole-plant phenotype assays

Based on previous studies, many plant species are reported to have early flowering phenotypes in response to environmental stresses (Wada and Takeno, 2010; Riboni et al., 2014). Therefore, we grew *C. campestris* on WT H1706 tomatoes and *CcLBD25* RNAi T1 transgenic tomato plants and then quantified how fast these *C. campestris* plants transitioned to their reproductive stage. The number of *C. campestris* plants that transitioned to the flowering stage was counted at 9, 10, and 14 DPA to test whether a stress-induced flowering phenotype could be observed.

To quantify the effect of *CcLBD25* downregulation on *C. campestris* growth, we infested 3-week-old tomato plants with ∼10 cm stem segments *C. campestris*, which were originally grown on WT H1706. We harvested all *C. campestris* tissues grown on WT H1706 and *CcLBD25* RNAi T2 transgenic plants at 14 DPA. These *C. campestris* tissues were then carefully separated from their host plant stems by hand, and their fresh weights were measured using chemical weighing scales.

### IVH system

Inspired by the previous discovery that *Cuscuta* haustoria can be induced by physical contact and far-red light signals (Tada et al., 1996), we developed an (IVH system for haustorium induction without hosts. In this method, we detached *Cuscuta* stem segments, which were right next to a stable haustorium attachment, from the *C. campestris* grown on WT plants and T2 *CcLBD25* RNAi transgenic plants. *Cuscuta* strands with shoot apices detached from a host plant were sandwiched between 3% Phytagel agar containing 0.5× Murashige and Skoog medium to provide tactile stimuli (Figure 6, A–C). These combined plates were then irradiated with far-red light for 2 h. After 5 d of growth in darkness in a 22°C growth chamber, prehaustoria were readily visible (Figure 6, D–E). We then counted the number of prehaustoria under a Zeiss SteREO Discovery, V12 microscope for quantification. Since the RNAi silencing signal is systemic (David-Schwartz et al., 2008; Alakonya et al., 2012)and IVH induction is rapid, we could validate the effect of *CcLBD25* RNAi on haustoria development. We are aware of a similar system that was reported recently (Kaga et al., 2020). However, we used two layers of agar gel instead of one layer of gel with one glass slide. This prevented prehaustoria from attaching to the glass slide, making it easier to detach prehaustoria for further analysis without damaging their structure. Second, we used far-red light instead of blue light irradiation. Both methods seem to be effective.

### Fresh tissue sectioning and histology

For fresh vibratome sections of haustoria attached to WT and *CcLBD25 RNAi* host stems, we collected samples and embedded them in 7% Plant Tissue Culture Agar. We then fixed these agar blocks in FAA (final concentration: 4% formaldehyde, 5% glacial acetic acid, and 50% ethanol) overnight, 50% ethanol for 1 h, and then transferred the samples to 70% ethanol for storage. These agar blocks were then sectioned using Lancer Vibratome Series 1000 to prepare 100 μm sections. We kept these sections in 4°C water and then conducted Toluidine Blue O Staining. We followed the published protocol (O'Brien et al., 1964) with some modifications. The sections were immersed in the stain for 30 s, and then washed with water three times for 30 s each wash. After removing the agar from around the sections using forceps, we mounted the sections with water on a slide and imaged using a Zeiss SteREO Discovery, V12 microscope, and a Nikon Eclipse E600 microscope.

## Code availability

Updated R scripts for MDS, PCA, and SOM analysis and GCN analysis are all deposited on GitHub (Link: https://github.com/MinYaoJhu/CcLBD25_project.git).

## Data availability

All data are available in the main text or the supplemental materials. LCM RNA-seq raw data are deposited on NCBI Sequence Read Archive (SRA) PRJNA687611.

## Accession numbers

LCM RNA-seq raw reads from this article can be found in the NCBI SRA data under accession number PRJNA687611.

## Supplemental data

The following materials are available in the online version of this article.

**Supplemental Figure S1**. MDS plot of expression profiles of all libraries from six different *C. campestris* tissue types mapped to the *C. campestris* genome.

**Supplemental Figure S2**. Heatmaps of gene expression profiles in z-scores for SOM2, SOM3, and SOM9 from *C. campestris* tissue type RNA-seq data mapped to *C. campestris* genome.

**Supplemental Figure S3**. *LBD25* phylogenetic tree with top significant sequences aligned with *CcLBD25*, *TcLBD25*, *AtLBD25*, and *SlLBD25*.

**Supplemental Figure S4**. MDS plot of RNA expression profile in all libraries from LCM of three different *C. campestris* developmental stages mapped to *C. campestris* genome.

**Supplemental Figure S5**. PCA analysis with SOM clustering and GCNs of gene expression in *C. campestris* haustoria across three developmental stages in LCM RNA-seq data.

**Supplemental Figure S6**. Heatmap of gene expression profiles in z-scores for SOM6 from *C. campestris* LCM RNA-seq data mapped to *C. campestris* genome.

**Supplemental Figure S7**. *CcLBD25* RNAi constructs for HIGS.

**Supplemental Figure S8**. Whole-plant phenotypes of *CcLBD25* RNAi transgenic tomato plants without *C. campestris* infestation treatment.

**Supplemental Figure S9**. Quantification of haustorium status on *CcLBD25* RNAi HIGS and WT plants.

**Supplemental Table S1**. The SOM clustering gene list in C. campestris tissue type RNA-seq data and results of PCA analysis and multilevel SOM clustering using selected genes with coefﬁcient of variation >0.85.

**Supplemental Table S2**. The gene list of SOM9 GCN modules from *C. campestris* tissue type RNA-seq data.

**Supplemental Table S3**. Combined annotation of *C. campestris* genes and transcriptome.

**Supplemental Table S4**. The GO enrichment results and statistics of SOM9 GCN modules from *C. campestris* tissue type RNA-seq data.

**Supplemental Table S5**. The gene list of SOM2, 3, 9 combined GCN modules from *C. campestris* tissue type RNA-seq data.

**Supplemental Table S6**. The GO enrichment results and statistics of SOM2, 3, 9 combined GCN modules from *C. campestris* tissue type RNA-seq data.

**Supplemental Table S7**. The SOM clustering gene list in LCM RNA-seq data and results of PCA analysis and multilevel SOM clustering using selected genes in the upper 50% quartile of coefﬁcient of variation.

**Supplemental Table S8**. The gene list in the modules of the GCN based on LCM RNA-seq expression with genes in tissue type RNA-seq SOM9.

**Supplemental Table S9**. The GO enrichment results and statistics of the modules of the GCN based on LCM RNA-seq expression with genes in tissue type RNA-seq SOM9.

**Supplemental Table S10**. Quantification and statistics of haustorium status on *CcLBD25* RNAi HIGS and WT plants by section ID.

**Supplemental Table S11**. Quantification and statistics of haustorium status on *CcLBD25* RNAi HIGS and WT plants by sample ID.

**Supplemental Table S12**. The primer pairs that are used for making the construct and quantifying expression level of *CcLBD25* by quantitative reverse transcriptase PCR (RT-qPCR).

**Supplemental Dataset S1**. Nucleotide sequence alignments with *CcLBD25*, *TcLBD25*, *AtLBD25*, *SlLBD25*, and their top 10 significant aligned sequences.

**Supplemental Dataset S2**. Sequence of the *CcLBD25* fragment that is used for making *CcLBD25* RNAi construct.

**Supplemental Dataset S3**. Sequence of the SUC2 promoter that is used for driving CcLBD25 RNAi construct in pTKOS.

## Supplementary Material

kiab231_Supplementary_Data

## Notes

Y.I. initiated the project and all work was done under consultation and supervision from N.R.S.; Y.I. wrote the original R scripts for MDS, PCA, and SOM analysis, and M.-Y.J. edited the scripts and applied them on further analyses; M.-Y.J. mapped the transcriptome data to *C. campestris* genome and performed SOM clustering and coexpression network analysis; M.F. made *CcLBD25* RNAi constructs and used the UC Davis transformation facility to generate transgenic plants; M.-Y.J. performed qPCR, functional characterization, and phenotyping of *CcLBD25* transgenic plants; M.-Y.J. designed the IVH system and conducted HIGS experiments and analyzed the data; M.F. conducted LCM to capture tissues and prepared libraries for RNA-seq; M.-Y.J. mapped the LCM RNA-seq data to *C. campestris* genome and performed SOM clustering and GCN analysis; M.Y.J. conducted phylogenetic analysis and verified *Cuscuta* species; C.W. analyzed haustorium connection status; M.-Y.J. wrote the initial manuscript and made figures and tables with primary editing from N.R.S.; N.R.S. supervised this project and serves as the author responsible for contact and communication.

The author responsible for distribution of materials integral to the findings presented in this article in accordance with the policy described in the Instructions for Authors (https://academic.oup.com/plphys/pages/general-instructions) is: Neelima R. Sinha (nrsinha@ucdavis.edu).
