# Supplementary material for: LATERAL ORGAN BOUNDARIES DOMAIN 25 functions as a key regulator of haustorium development in dodders
Source: Plant Physiol. 2021 May 27;186(4):2093–110. doi: 10.1093/plphys/kiab231 (PMC8331169; doi:10.1093/plphys/kiab231)
Supplement: kiab231_Supplementary_Data [file kiab231_supplementary_data.zip › pp.01781.2020-s02.pdf]

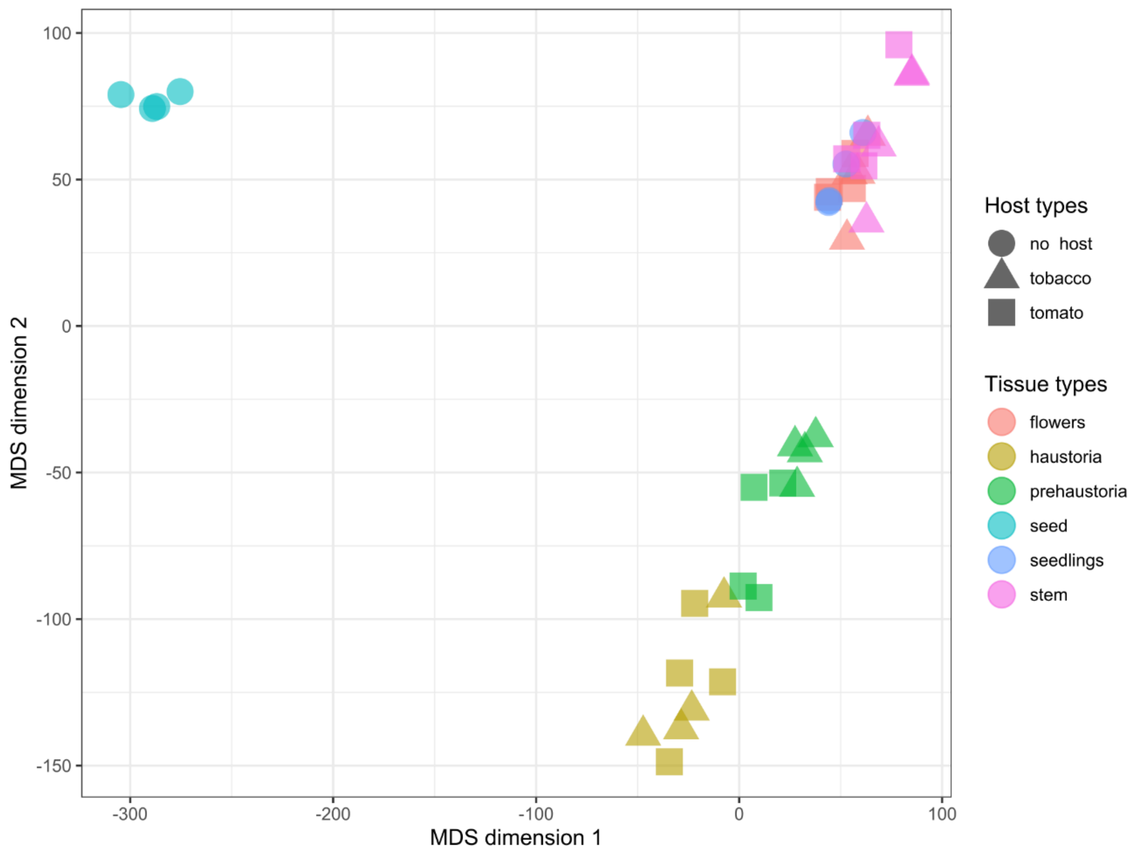

Supplemental Figure S1. Multidimensional scaling (MDS) plot of expression profiles of all libraries from 6 different *C. campestris* tissue types mapped to the *C. campestris* genome. Stem, prehaustorium, haustorium, and flower tissue types have 8 libraries for each. Triangles represent the 4 libraries using tissue collected from *C. campestris* grown on *N. benthamiana*. Squares represent the 4 libraries using tissue collected from *C. campestris* grown on *S. lycoperscum*. Seed and seedling tissue types have 4 libraries for each. Circles represent seed and seedling libraries that are not dependent on hosts.

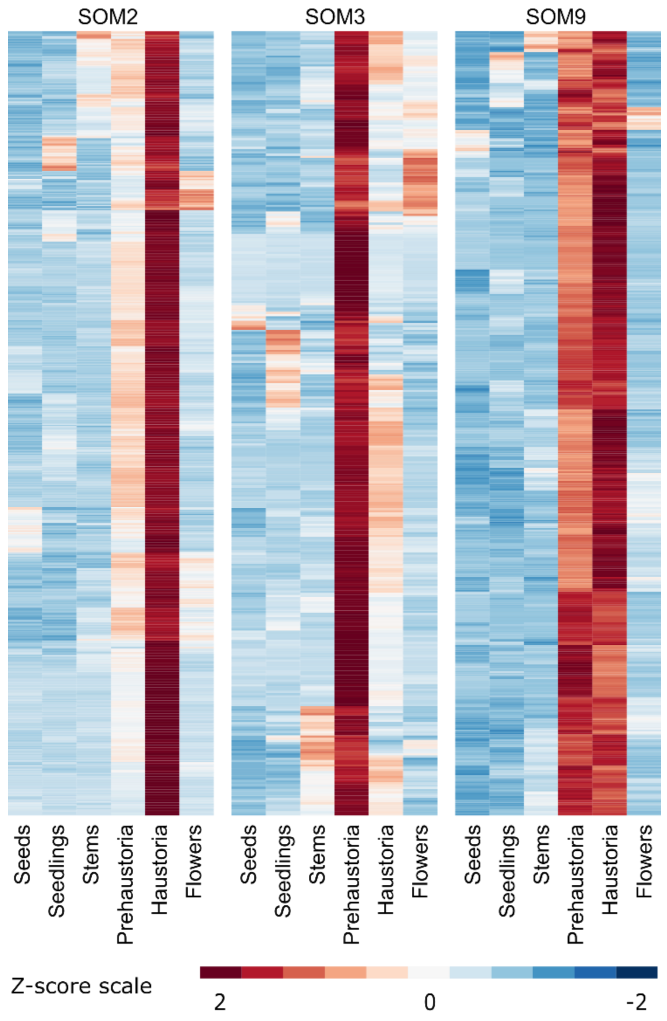

Supplemental Figure S2. Heatmaps of gene expression profiles in z-scores for SOM2, SOM3, and SOM9 from *C. campestris* tissue type RNA-Seq data mapped to *C. campestris* genome. The complete gene lists for all SOM units with SOM distances and PCA principal component values are included in Supplemental Table S1.

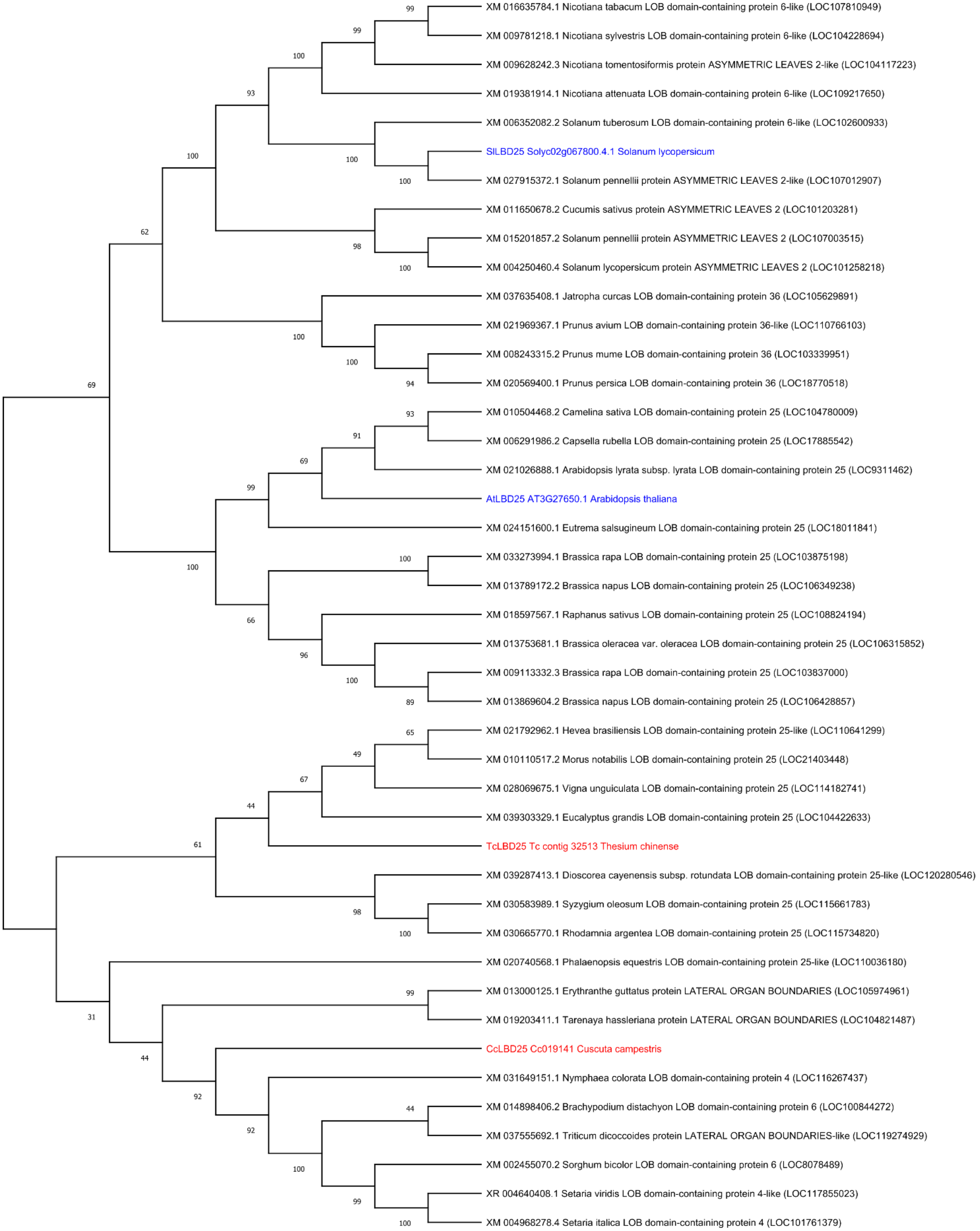
XM 016635784.1 Nicotiana tabacum LOB domain-containing protein 6-like (LOC107810949)
XM 009781218.1 Nicotiana sylvestris LOB domain-containing protein 6-like (LOC104228694)
XM 009628242.3 Nicotiana tomentosiformis protein ASYMMETRIC LEAVES 2-like (LOC104117223)
XM 019381914.1 Nicotiana attenuata LOB domain-containing protein 6-like (LOC109217650)
XM 006352082.2 Solanum tuberosum LOB domain-containing protein 6-like (LOC102600933)
SlLBD25 Solyc02g067800.4.1 Solanum lycopersicum
XM 027915372.1 Solanum pennellii protein ASYMMETRIC LEAVES 2-like (LOC107012907)
XM 011650678.2 Cucumis sativus protein ASYMMETRIC LEAVES 2 (LOC101203281)
XM 015201857.2 Solanum pennellii protein ASYMMETRIC LEAVES 2 (LOC107003515)
XM 004250460.4 Solanum lycopersicum protein ASYMMETRIC LEAVES 2 (LOC101258218)
XM 037635408.1 Jatropha curcas LOB domain-containing protein 36 (LOC105629891)
XM 021969367.1 Prunus avium LOB domain-containing protein 36-like (LOC110766103)
XM 008243315.2 Prunus mume LOB domain-containing protein 36 (LOC103339951)
XM 020569400.1 Prunus persica LOB domain-containing protein 36 (LOC18770518)
XM 010504468.2 Camelina sativa LOB domain-containing protein 25 (LOC104780009)
XM 006291986.2 Capsella rubella LOB domain-containing protein 25 (LOC17885542)
XM 021026888.1 Arabidopsis lyrata subsp. lyrata LOB domain-containing protein 25 (LOC9311462)
AtLBD25 AT3G27650.1 Arabidopsis thaliana
XM 024151600.1 Eutrema salsugineum LOB domain-containing protein 25 (LOC18011841)
XM 033273994.1 Brassica rapa LOB domain-containing protein 25 (LOC103875198)
XM 013789172.2 Brassica napus LOB domain-containing protein 25 (LOC106349238)
XM 018597567.1 Raphanus sativus LOB domain-containing protein 25 (LOC108824194)
XM 013753681.1 Brassica oleracea var. oleracea LOB domain-containing protein 25 (LOC106315852)
XM 009113332.3 Brassica rapa LOB domain-containing protein 25 (LOC103837000)
XM 013869604.2 Brassica napus LOB domain-containing protein 25 (LOC106428857)
XM 021792962.1 Hevea brasiliensis LOB domain-containing protein 25-like (LOC110641299)
XM 010110517.2 Morus notabilis LOB domain-containing protein 25 (LOC21403448)
XM 028069675.1 Vigna unguiculata LOB domain-containing protein 25 (LOC114182741)
XM 039303329.1 Eucalyptus grandis LOB domain-containing protein 25 (LOC104422633)
TcLBD25 Tc contig 32513 Thesium chinense
XM 039287413.1 Dioscorea cayenensis subsp. rotundata LOB domain-containing protein 25-like (LOC120280546)
XM 030583989.1 Syzygium oleosum LOB domain-containing protein 25 (LOC115661783)
XM 030665770.1 Rhodamnia argentea LOB domain-containing protein 25 (LOC115734820)
XM 020740568.1 Phalaenopsis equestris LOB domain-containing protein 25-like (LOC110036180)
XM 013000125.1 Erythranthe guttatus protein LATERAL ORGAN BOUNDARIES (LOC105974961)
XM 019203411.1 Tarenaya hassleriana protein LATERAL ORGAN BOUNDARIES (LOC104821487)
CcLBD25 Cc019141 Cuscuta campestris
XM 031649151.1 Nymphaea colorata LOB domain-containing protein 4 (LOC116267437)
XM 014898406.2 Brachypodium distachyon LOB domain-containing protein 6 (LOC100844272)
XM 037555692.1 Triticum dicoccoides protein LATERAL ORGAN BOUNDARIES-like (LOC119274929)
XM 002455070.2 Sorghum bicolor LOB domain-containing protein 6 (LOC8078489)
XR 004640408.1 Setaria viridis LOB domain-containing protein 4-like (LOC117855023)
XM 004968278.4 Setaria italica LOB domain-containing protein 4 (LOC101761379)

Supplemental Figure S3. *LBD25* phylogenetic tree with top significant sequences aligned with *CcLBD25*, *TcLBD25*, *AtLBD25* and *SlLBD25*. The top 10 significant aligned sequences were obtained using Blastn based on *CcLBD25*, *TcLBD25*, *AtLBD25* and *SlLBD25* nucleotide sequences, respectively. The evolutionary relationships were inferred using the Maximum Likelihood method and Tamura-Nei model. The bootstrap consensus tree was built from 500 replicates. Evolutionary analysis was conducted in MEGA X. *CcLBD25* and *TcLBD25* are labeled in red. *AtLBD25* and *SlLBD25* are labeled in blue. The sequence alignment that is used for building this gene phylogenetic tree is included in Supplemental Data S1.

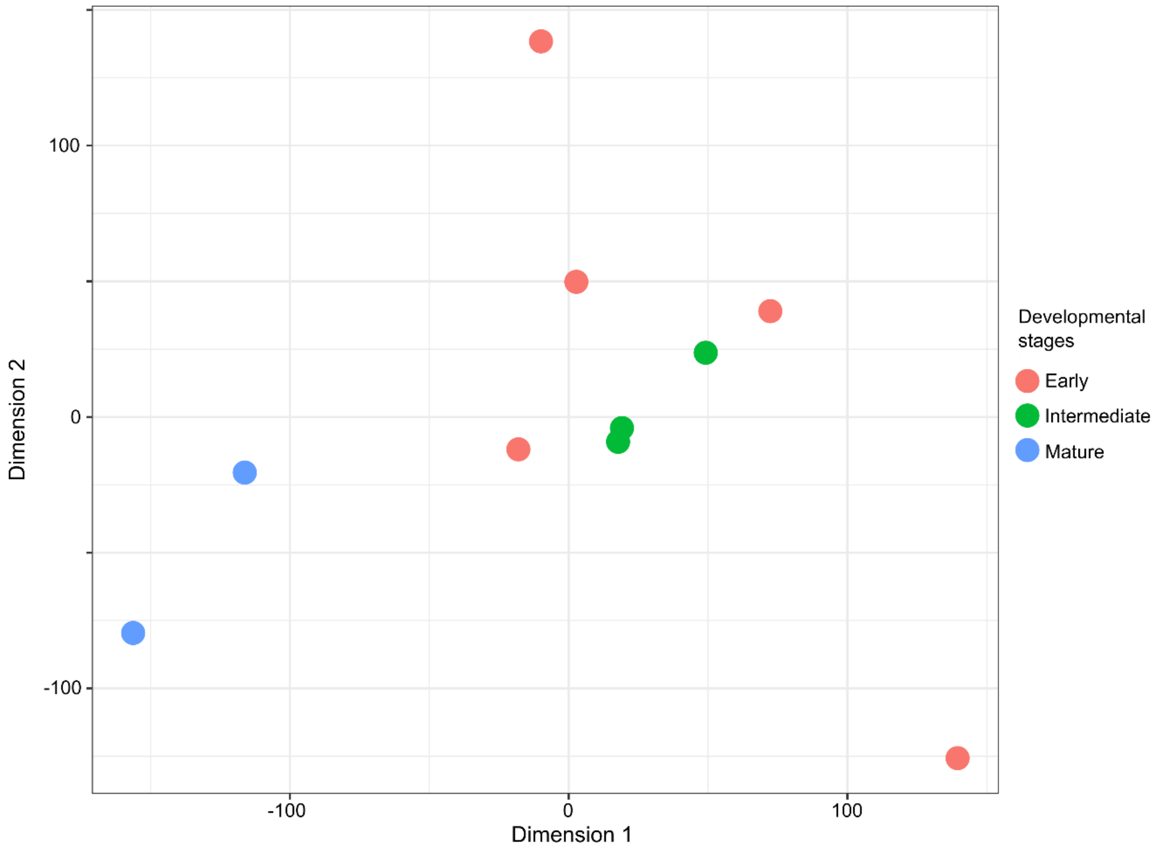

Supplemental Figure S4. MDS plot of RNA expression profile in all libraries from LCM of three different *C. campestris* developmental stages mapped to *C. campestris* genome. Early-stage has 5 libraries. Intermediate-stage has 3 libraries. Mature-stage has 2 libraries.

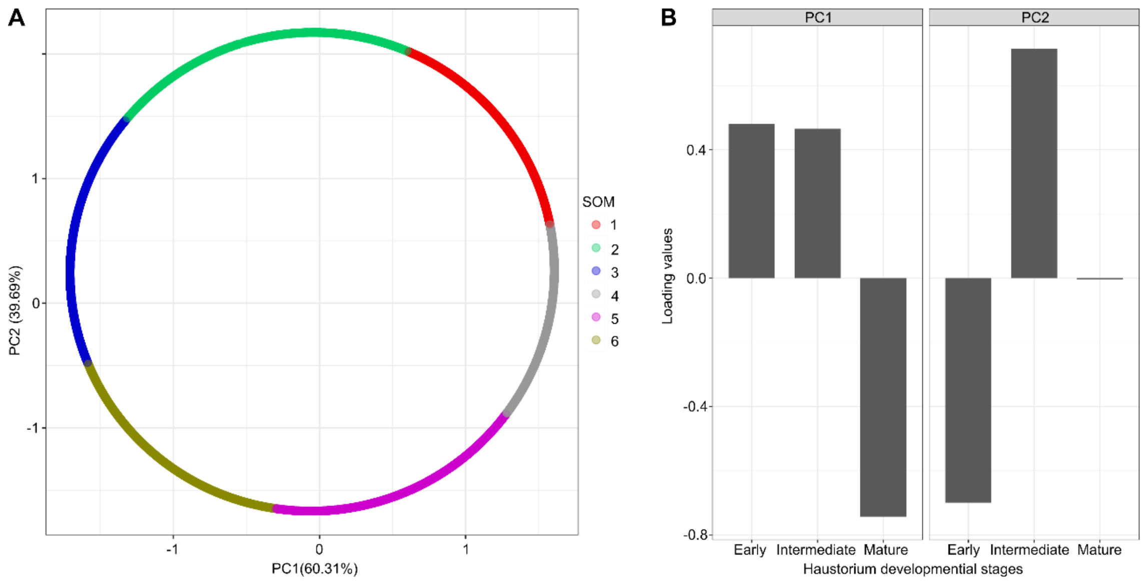

Supplemental Figure S5. PCA analysis with SOM clustering and GCNs of gene expression in *C. campestris* haustoria across three developmental stages in LCM RNA-Seq data. (A) PCA analysis based on gene expression pattern across three developmental stages, early, intermediate, and mature. Each dot represents a gene and is in the color indicating their corresponding SOM groups. (B) Loading values of PC1 and PC2. PC1 separates the genes that are specifically expressed in mature-stage from those that are expressed in the early and intermediate-stages. PC2 divides the early-stage-specific genes from intermediate-stage-specific genes.

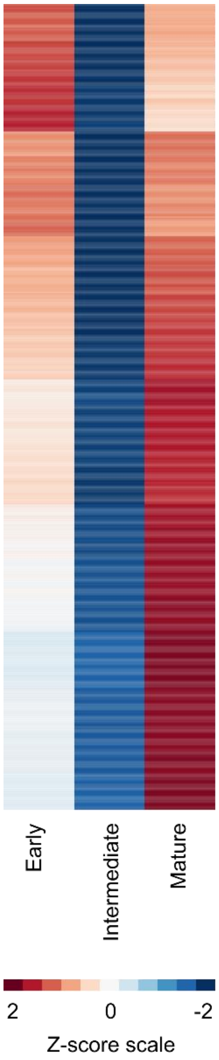

Supplemental Figure S6. Heatmap of gene expression profiles in z-scores for SOM6 from *C. campestris* LCM RNA-Seq data mapped to *C. campestris* genome.

A

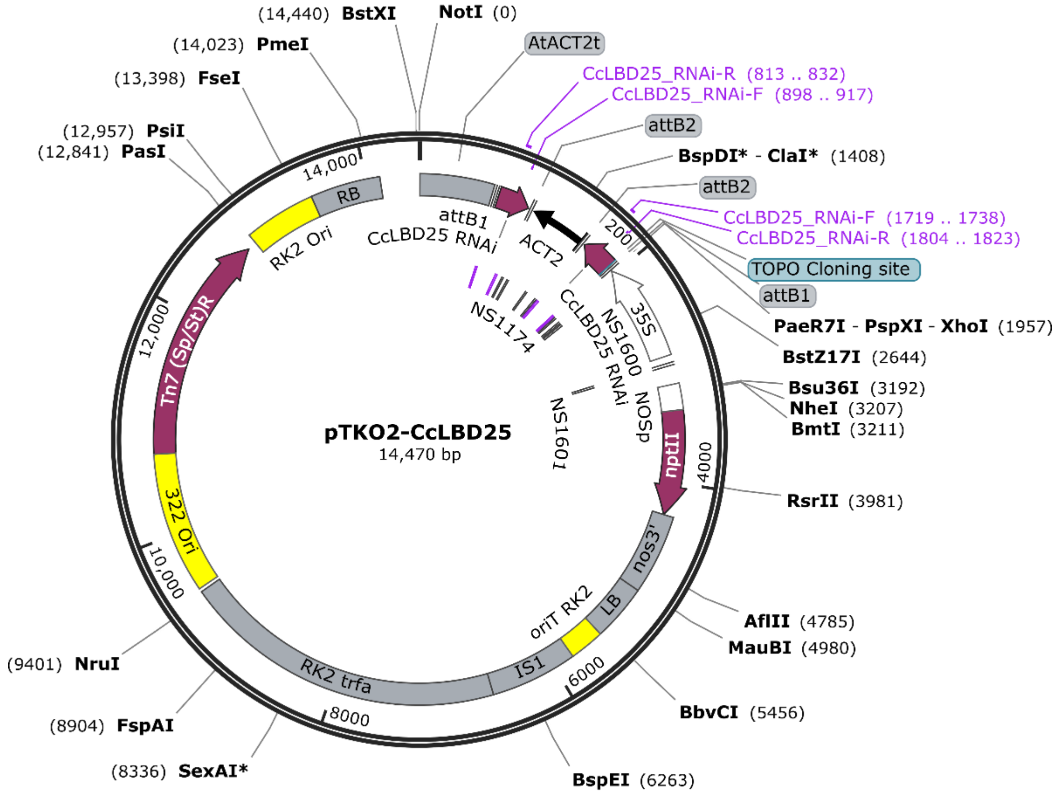
pTKO2-CcLBD25
14,470 bp
(14,440) BstXI
NotI (0)
(14,023) PmeI
(13,398) FseI
(12,957) PsiI
(12,841) PasI
AtACT2t
CcLBD25_RNAi-R (813 .. 832)
CcLBD25_RNAi-F (898 .. 917)
attB2
BspDI* - ClaI* (1408)
attB2
CcLBD25_RNAi-F (1719 .. 1738)
CcLBD25_RNAi-R (1804 .. 1823)
TOPO Cloning site
attB1
PaeR7I - PspXI - XhoI (1957)
BstZ17I (2644)
Bsu36I (3192)
NheI (3207)
BmtI (3211)
RsrII (3981)
AflII (4785)
MauBI (4980)
BbvCI (5456)
BspEI (6263)
(8336) SexAI*
(8904) FspAI
(9401) NruI
RB
RK2 Ori
attB1
CcLBD25 RNAi
ACT2
35S
NS1174
CcLBD25 RNAi
NS1600
NOSp
NS1601
nptII
nos3'
LB
oriT RK2
IS1
RK2 trfa
322 Ori
Tn7 (Sp/St)R
14,000
12,000
10,000
8000
6000
4000
2000

B

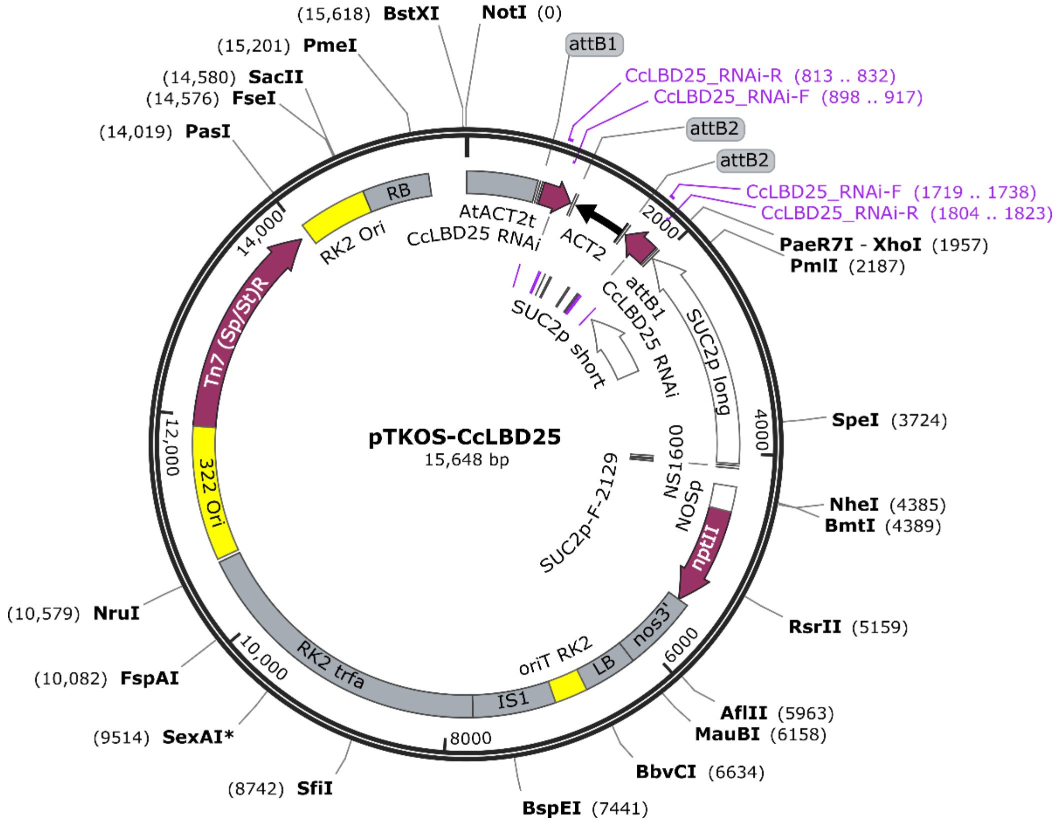
pTKOS-CcLBD25
15,648 bp
(15,618) BstXI
NotI (0)
(15,201) PmeI
(14,580) SacII
(14,576) FseI
(14,019) PasI
attB1
CcLBD25_RNAi-R (813 .. 832)
CcLBD25_RNAi-F (898 .. 917)
attB2
attB2
CcLBD25_RNAi-F (1719 .. 1738)
CcLBD25_RNAi-R (1804 .. 1823)
PaeR7I - XhoI (1957)
PmlI (2187)
SpeI (3724)
NheI (4385)
BmtI (4389)
RsrII (5159)
AflII (5963)
MauBI (6158)
BbvCI (6634)
BspEI (7441)
(8742) SfiI
(9514) SexAI*
(10,082) FspAI
(10,579) NruI
RB
RK2 Ori
AtACT2t
CcLBD25 RNAi
ACT2
attB1
CcLBD25 RNAi
SUC2p short
SUC2p long
NS1600
SUC2p-F-2129
NOSp
nptII
nos3'
LB
oriT RK2
IS1
RK2 trfa
322 Ori
Tn7 (Sp/St)R
14,000
12,000
10,000
8000
6000
4000
2000

Supplemental Figure S7. *CcLBD25* RNAi constructs for host-induced gene silencing (HIGS). (A) pTKO2 has 35S promoter to drive *CcLBD25* RNAi construct. (B) pTKOS has SUC2 promoter to drive *CcLBD25* RNAi construct. The segment of CcLBD25 sequence used for RNAi construct is included in Supplemental Data S2. The complete SUC2 promoter sequence is included in Supplemental Data S3.

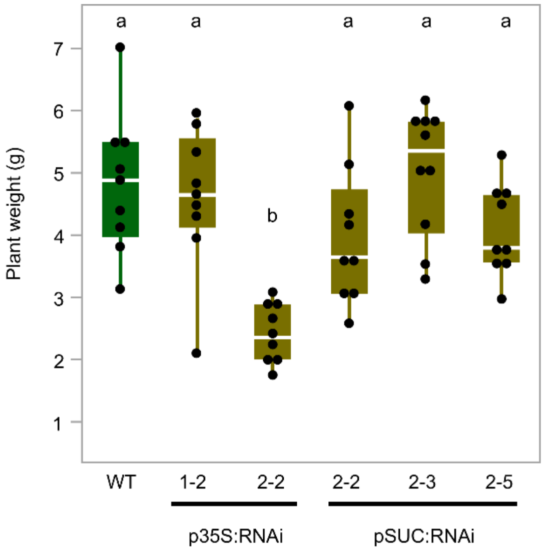

Plant weight (g)
1
2
3
4
5
6
7
a
a
b
a
a
a
WT
1-2
2-2
2-2
2-3
2-5
p35S:RNAi
pSUC:RNAi

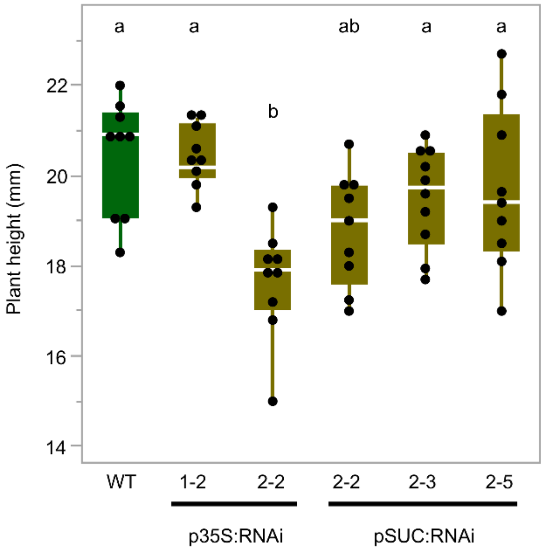

Plant height (mm)
14
16
18
20
22
a
a
b
ab
a
a
WT
1-2
2-2
2-2
2-3
2-5
p35S:RNAi
pSUC:RNAi

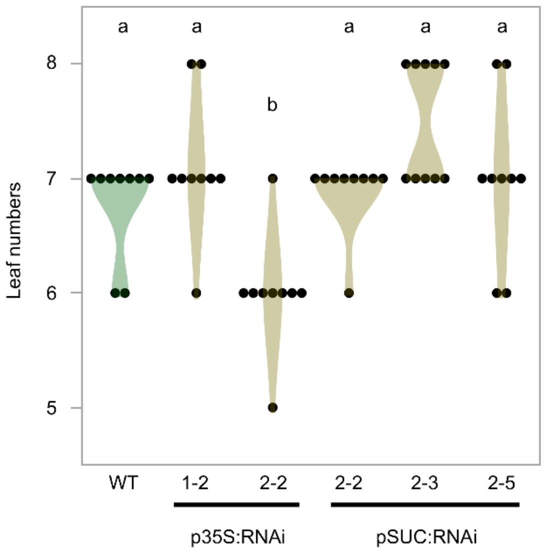

Leaf numbers
5
6
7
8
a
a
b
a
a
a
WT
1-2
2-2
2-2
2-3
2-5
p35S:RNAi
pSUC:RNAi

Supplemental Figure S8. Whole-plant phenotypes of *CcLBD25* RNAi transgenic tomato plants without *C. campestris* infestation treatment. (A) Comparison of 4-week-old tomato plant biomass (above ground fresh weight). Data are presented in grams. (B) Comparison of 4-week-old tomato plant height. Data are presented in centimeters. (C) Comparison of 4-week-old tomato plant leaf number. (A-C) Data presented are assessed using pair-wise comparisons with the Tukey test. P-value of the contrasts between “a” and “b” is less than 0.05.

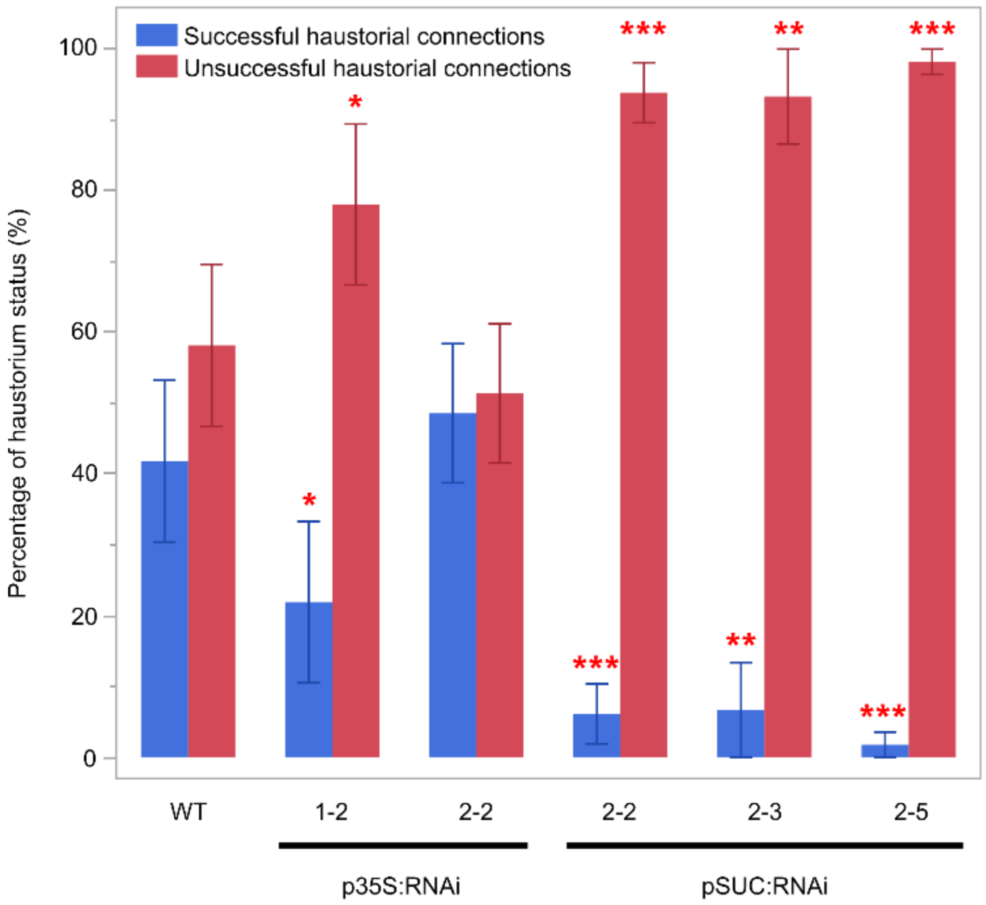

Successful haustorial connections
Unsuccessful haustorial connections
Percentage of haustorium status (%)
0
20
40
60
80
100
*
*
***
***
**
**
***
***
WT
1-2
2-2
2-2
2-3
2-5
p35S:RNAi
pSUC:RNAi

Supplemental Figure S9. Quantification of haustorium status on *CcLBD25* RNAi HIGS and wild-type plants. p35S:RNAi indicates the transgenic plants with the 35S promoter driving *CcLBD25* RNAi construct. pSUC:RNAi indicates the transgenic plants with the SUC2 promoter driving *CcLBD25* RNAi construct. Successful haustorial connections include the haustoria that formed vascular connections with the host or the haustoria with searching hyphae penetrated into the host cortex. Unsuccessful haustorial connections include the haustoria penetrated into the host cortex but without searching hyphae, or the haustoria that are only attached on the host stem surface. Data presented are assessed using one-tailed Welch's t-test with wild-type (WT) as control. "*" p-value < 0.13. "**" p-value < 0.05. "***" p-value < 0.01. Sample size: H1706, 136 sections from 8 biological replicates; p35S:RNAi 1-2, 55 sections from 4 biological replicates; p35S:RNAi 2-2, 105 sections from 4 biological replicates; pSUC:RNAi 2-2, 103 sections from 8 biological replicates; pSUC:RNAi 2-3, 69 sections from 7 biological replicates; pSUC:RNAi 2-5, 12 sections from 7 biological replicates. Complete quantification tables by sections and by samples are included in Supplemental Table S11 and S12.
